# Gut Microbiota Taxon-Dependent Transformation of Microglial M1/M2 Phenotypes Underlying Mechanisms of Spatial Learning and Memory Impairment after Chronic Methamphetamine Exposure

**DOI:** 10.1128/spectrum.00302-23

**Published:** 2023-05-22

**Authors:** Yulong Wu, Zhouyan Dong, Xinze Jiang, Lei Qu, Wei Zhou, Xu Sun, Jiangshan Hou, Hongmei Xu, Mei Cheng

**Affiliations:** a Department of Pathogenic Biology, Binzhou Medical University, Yantai, China; b Department of Health and Disease Management, Binzhou Medical University, Yantai, China; Jilin University

**Keywords:** microbiota perturbation, methamphetamine, microglial phenotypes, BDNF, learning and memory impairment, microbiota dysbiosis

## Abstract

Methamphetamine (METH) exposure may lead to cognitive impairment. Currently, evidence suggests that METH exposure alters the configuration of the gut microbiota. However, the role and mechanism of the gut microbiota in cognitive impairment after METH exposure are still largely unknown. Here, we investigated the impact of the gut microbiota on the phenotype status of microglia (microglial phenotypes M1 and microglial M2) and their secreting factors, the subsequent hippocampal neural processes, and the resulting influence on spatial learning and memory of chronically METH-exposed mice. We determined that gut microbiota perturbation triggered the transformation of microglial M2 to M1 and a subsequent change of pro-brain-derived neurotrophic factor (proBDNF)–p75^NTR^–mature BDNF (mBDNF)–TrkB signaling, which caused reduction of hippocampal neurogenesis and synaptic plasticity-related proteins (SYN, PSD95, and MAP2) and, consequently, deteriorated spatial learning and memory. More specifically, we found that *Clostridia*, *Bacteroides*, *Lactobacillus*, and *Muribaculaceae* might dramatically affect the homeostasis of microglial M1/M2 phenotypes and eventually contribute to spatial learning and memory decline after chronic METH exposure. Finally, we found that fecal microbial transplantation could protect against spatial learning and memory decline by restoring the microglial M1/M2 phenotype status and the subsequent proBDNF-p75^NTR^/mBDNF-TrkB signaling in the hippocampi of chronically METH-exposed mice.

**IMPORTANCE** Our study indicated that the gut microbiota contributes to spatial learning and memory dysfunction after chronic METH exposure, in which microglial phenotype status plays an intermediary role. The elucidated “specific microbiota taxa-microglial M1/M2 phenotypes-spatial learning and memory impairment” pathway would provide a novel mechanism and elucidate potential gut microbiota taxon targets for the no-drug treatment of cognitive deterioration after chronic METH exposure.

## INTRODUCTION

Methamphetamine (METH), a synthetic amphetamine-type drug, has become one of the most commonly used illicit drugs worldwide (1). METH causes brain damage, giving rise to neurological and psychiatric dysfunction (2), such as cognitive impairment (3, 4). A number of research studies have shown that neurodegeneration associated with METH exposure may be sustained for an extended period (5, 6). Therefore, treating METH-induced neurotoxic damage has become an urgent health concern. However, there is still a lack of state-approved pharmacological intervention to treat cognition deficiency after METH exposure, and the efficiency of traditional behavioral-cognitive therapy is barely satisfactory. Therefore, it is essential to explore the underlying mechanism of METH exposure-associated cognitive dysfunction and thereby develop alternative therapeutic strategies for cognitive deterioration after METH exposure.

It was recently revealed that exposure to METH significantly changes the diversity and composition of the gut microbiota (7, 8). Specifically, METH treatment downregulated the abundance of probiotics producing short-chain fatty acids (SCFAs) (9) while elevating the conditionally pathogenic bacteria responsible for intestinal inflammation (9, 10). Growing evidence has shown that gut microbiota perturbations are compellingly correlated with neurological diseases through the microbiota-gut-brain axis (11, 12). METH increases the permeability of the blood-brain barrier (BBB) via downregulation of tight junction proteins (13), making it possible for members of the intestinal microbiota and their products to enter the brain. Therefore, we speculate that METH-associated perturbation of the intestinal microbiota may act as a mediator in the communication between the gut and the brain, and restoring the gut microbiota’s homeostasis may constitute a novel therapy for cognitive deficiency that occurs after chronic METH exposure.

Recent studies determined that the gut microbiota pivotally influences cellular metabolism and maintains the maturation and function of microglia (14, 15). Under normal physiological circumstances, microglia are vital for synaptic pruning and remodeling in the brain through neuron-microglia signaling during development and adulthood (16, 17). Under neuroinflammation conditions, microglia are the resident macrophages of the central nervous system and act as a primary active immune defense (18). Many lines of evidence have indicated that microglia dysfunction contributes to neurological diseases such as Parkinsons disease (PD), Alzheimer’s disease (AD), and depression (19–21). Notably, the communication between microglia and neurons is bidirectional, depending on the status of the microglial phenotype, which is regulated by the brain’s microenvironment (22). The classically activated M1 microglia trigger safeguard against infection and is deemed to promote proinflammatory. It has been demonstrated that microglial activation and the secretion of proinflammatory cytokines (e.g., interleukin 1β [IL-1β], IL-6, and tumor necrosis factor alpha [TNF-α]) play a critical role in the neuroinflammation and subsequent neural damage after METH exposure (23, 24). Conversely, the alternatively activated M2 microglia usually exert remedial effects during excess neuroinflammation by secreting anti-inflammatory cytokines (e.g., IL-4, IL-10, and transforming growth factor beta [TGF-β]) and neurotrophic factors such as brain-derived neurotrophic factor (BDNF) (25). Microglial BDNF is critical for neuron-microglia cross talk, where BDNF binds high-affinity receptors of dendritic spines involved in synapse plasticity, which is associated with cognitive function (25). BDNF can be synthesized from a precursor protein called proBDNF and secreted as a mixture of mature BDNF (mBDNF) and proBDNF. In our previous study, we found that BDNF underlies the pathogenesis of METH exposure-induced neurodysfunction (26). However, it remains unclear whether and how the transformation of microglial M1/M2 phenotype and the subsequent changes of the M2-derived BDNF signaling system are involved in the association of gut microbiota perturbation and cognitive deficiency after METH exposure.

In the present study, we investigated the characteristic composition of gut microbiotas and spatial learning and memory of chronically METH-exposed mice. Moreover, we examined the status of microglial M1/M2 phenotypes and their respective secreted factors (neuroinflammatory cytokines and molecules of the BDNF signaling system), as well as subsequent hippocampal neurogenesis and synaptic plasticity, to elucidate the mechanisms of spatial learning and memory impairment induced by gut microbiota perturbation after chronic METH exposure. Furthermore, we identified specific gut microbiota taxa and assessed the mediation effect sizes of hippocampal microglial M1/M2 phenotypes between these taxa and spatial learning and memory impairment. Finally, we elucidated the therapeutic impact of fecal transplants on microglial M1/M2 phenotype homeostasis and on spatial learning and memory of chronically METH-exposed mice. This may not only help us to understand the causative role of the gut microbiota in learning and memory deterioration after chronic METH exposure but also prompt novel therapeutic approaches targeting the intestinal microbiota to relieve METH-exposure-associated neurotoxicity.

## RESULTS

### Chronic METH exposure induced gut microbiome perturbation and spatial learning and memory impairment.

**FIG 1:**
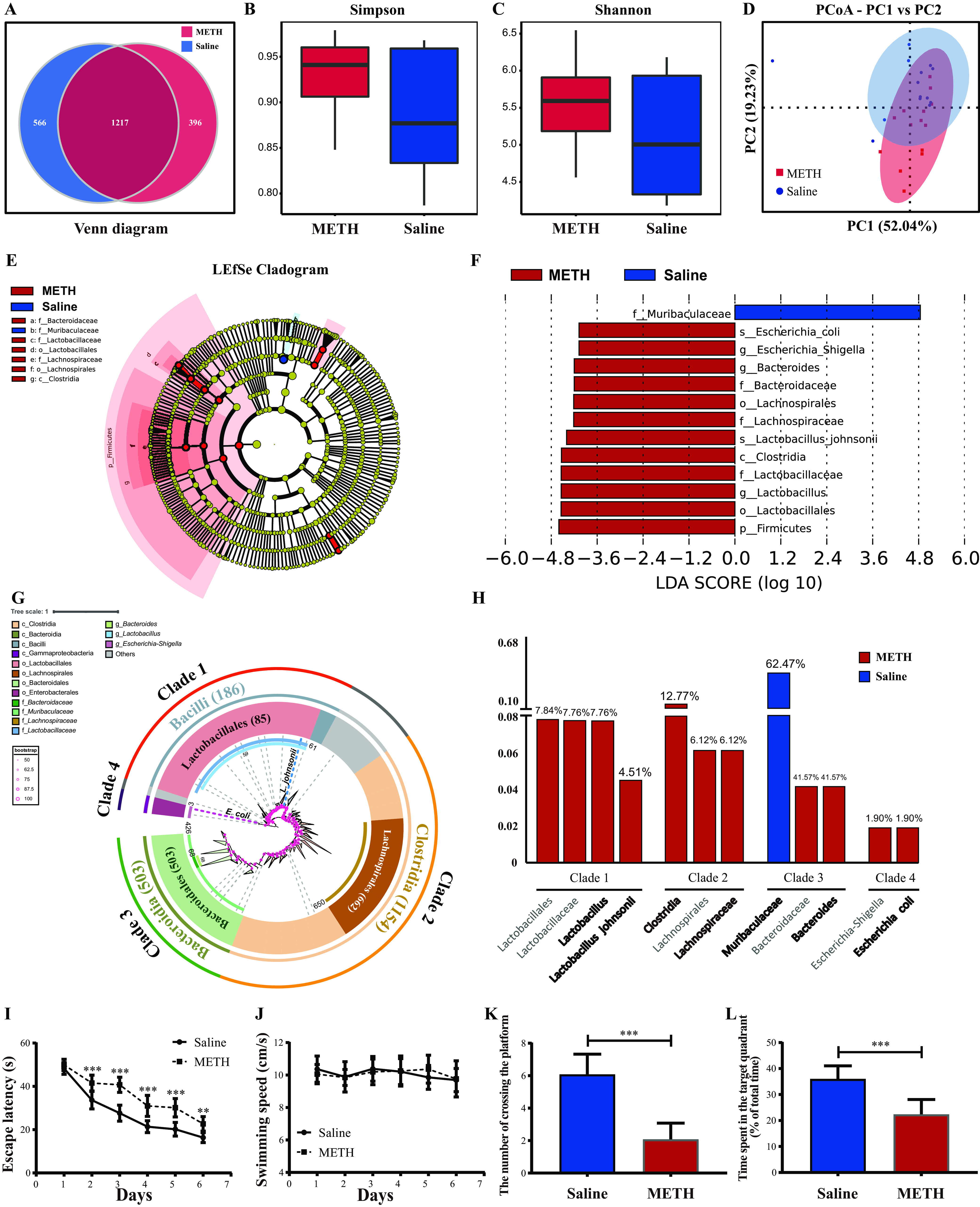
Chronic METH exposure induced gut microbiome dysbiosis and spatial learning and memory impairment. (A) Venn diagram of unique and shared OTUs in the METH and saline groups. (B and C) Simpson and Shannon indexes show the difference in alpha diversity between the two groups. (D) PCoA plot based on weighted UniFrac distances, demonstrating significant changes in the gut microbiota after chronic METH exposure. Axis 1 (PC1) explained 52.04% of the variation, and axis 2 (PC2) explained 19.23% of the variation. (E) LEfSe cladogram. The blue and red colors represent distinct taxa in the saline and METH groups, respectively, whereas yellow indicates no significant differences between the groups. (F) LDA value distribution histogram. Taxa with LDA values greater than 4 are presented. (G) Evolutionary relationships of characteristic taxa depicted with a maximum-likelihood phylogenetic tree generated by IQtree software and visualized with iTOL. Numbers are the numbers of OTUs belonging to each taxon. (H) Relative abundance histogram of distinct taxa. (I and J) Escape latency and the swimming speed during the place navigation of the MWM test showed that spatial learning ability was impaired after chronic METH exposure. (K and L) Numbers of times crossing the platform from a previously determined spot and time spent in the target quadrant (percent of total time) during the spatial probe of the MWM test indicated that spatial memory ability was impaired after chronic METH exposure. Data are means and SEM (*n* = 12 to 14). **, *P* < 0.01; ***, *P* < 0.001.

To elucidate the alteration of the gut microbiome after chronic METH exposure, 16S rRNA gene sequencing was performed on fecal samples. A Venn diagram showed that a total of 2,179 operational taxonomic units (OTUs) were acquired, of which 1,217 were shared between the METH and saline groups; 566 and 396 OTUs were unique in the saline group and the METH group, respectively (Fig. 1A). Alpha diversity, determined by Simpson and Shannon indexes, demonstrated that the abundance and diversity of the gut microbiota declined in the METH group compared to the saline group (Fig. 1B and C). Principal-coordinate analysis (PCoA) results showed the distinct separation of the METH and saline groups (Fig. 1D) (ADONIS test: *R*^2^ = 0.11, *P* < 0.01). Linear discriminant analysis (LDA) effect size (LEfSe) was used to differentiate the two groups. The cladogram (Fig. 1E) and LDA value distribution histogram (Fig. 1F) revealed the discriminant taxa enriched in the saline group (blue) and the METH group (red). *Firmicutes* (phylum), *Clostridia* (class), *Lachnospirales* (order), *Lactobacillales* (order), *Bacteroidaceae* (family), *Lachnospiraceae* (family), *Lactobacillaceae* (family), Escherichia-*Shigella* (genus), *Bacteroides* (genus), *Lactobacillus* (genus), Escherichia coli (species), and Lactobacillus johnsonii (species) were detected as discriminant taxa enriched in the METH group, whereas *Muribaculaceae* (family) was the discriminant taxon enriched in the saline group. The phylogenetic relationships and relative abundances among the above-mentioned taxa are illustrated in Fig. 1G and H, respectively.

METH exposure has deleterious effects on cognition (27–29). In the present research, we detected spatial learning and memory ability by using the Morris water maze (MWM). The place navigation test results on days 1 to 6 are shown in Fig. 1I and J. With the increase in training days, all groups gradually reduced the escape latency required to locate the hidden platform. There was a significant interaction between groups and times [*F*(5, 110) = 7.855, *P* < 0.001] with regard to escape latency. *Post hoc* analyses showed that the escape latency of the METH group was longer than that of the saline group from the 2nd day to the 6th day. For the swimming speed, no significant differences were observed between the two groups as the training days progressed (Fig. 1J). These results indicated that chronic METH exposure impaired spatial learning ability. As shown in Fig. 1K and L, the results of the spatial probe trial on the 7th day showed that mice in the METH group crossed the platform less often and spent a shorter time in the target quadrant than mice in the saline group. These results demonstrated that METH chronic exposure impaired spatial memory ability.

### Chronic METH exposure altered the gut microbiota metabolome.

**FIG 2:**
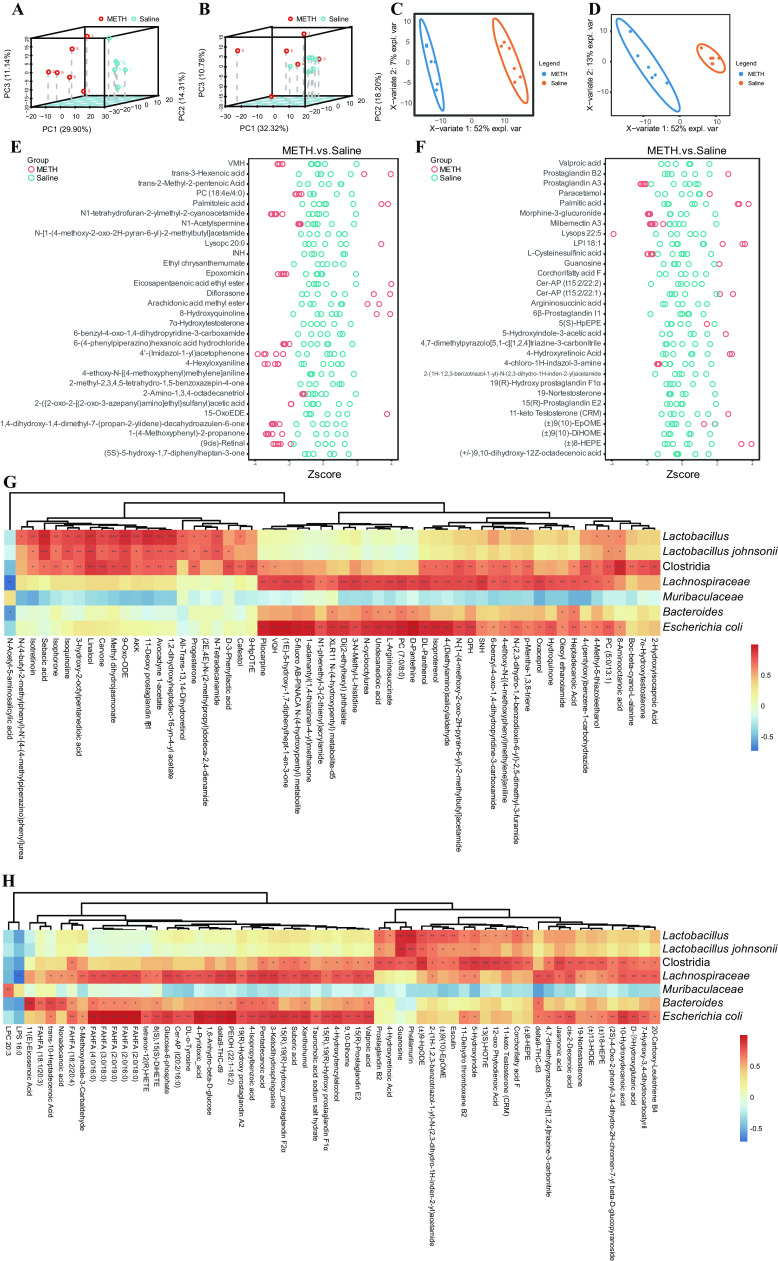
METH exposure altered the gut microbiota metabolome. (A) PCA plot of fecal metabolites in ES^+^. (B) PCA plot of fecal metabolites in ES^−^. (C) PLS-DA analysis of fecal metabolites in ES^+^. (D) PLS-DA analysis of fecal metabolites in ES^−^. (E) Different fecal metabolites between the METH and saline group in ES^+^ (top 30 results). (F) Different fecal metabolites between the METH and saline group in ES^−^ (top 30 results). (G) Metabolites which showed significant correlation with critical microbiota taxa in ES^+^. (H) Metabolites which showed significant correlation with critical microbiota taxa in ES^−^. *n* = 6 for each group. *, *P* < 0.05; **, *P* < 0.01; ***, *P* < 0.001.

In total, 314 and 176 significantly different metabolites were detected in electrospray positive-ion mode (ES^+^) and electrospray negative-ion mode (ES^−^), respectively. Principal-component analysis (PCA) (Fig. 2A and B) and partial least-squares discriminant analysis (PLS-DA) showed that the overall fecal metabolite profiles differed in the two groups (Fig. 2C and D). Compared with the saline group, prostaglandin A3, morphine-3-glucuronide, milbemectin A3, l-cysteinesulfinic acid, and 4-chloro-1H-indazol-3-amine were increased in ES^−^; meanwhile, Val-Met-His [VMH], 1,2-diacyl-sn-glycero-3-phosphocholine (PC) (18:4e/4:0), N1-tetrahydrofuran-2-ylmethyl-2-cyanoacetamide, N1-acetylspermine, epoxomicin, etc., were increased in ES^+^ after chronic METH treatment (Fig. 2E and F). The enrichment analysis revealed that several metabolic pathways were enriched in the METH group both in ES^+^ and ES^−^ (see Fig. S1 in in the supplemental material), including lipid metabolism, amino acid metabolism, fatty acids, conjugates, etc.

Based on the 16S rRNA gene sequencing, *Lactobacillus*, Lactobacillus johnsonii, *Clostridia*, *Lachnospiraceae*, *Muribaculaceae*, *Bacteroides*, and Escherichia coli were chosen as key microbiota taxa, and Fig. 2G and H display the metabolites with significant association (*P* < 0.05). For example, *Lactobacillus* and Lactobacillus johnsonii are significantly positively correlated with all-trans-13,14-dihydroretinol, 11-deoxy prostaglandin F1β, 13(S)-HOTrE, etc. These results provided potential target metabolites of the key microbiota taxa.

### Chronic METH exposure elevated the plasma and hippocampal LPS levels in parallel with the intestinal and BBB permeability disruption.

**FIG 3:**
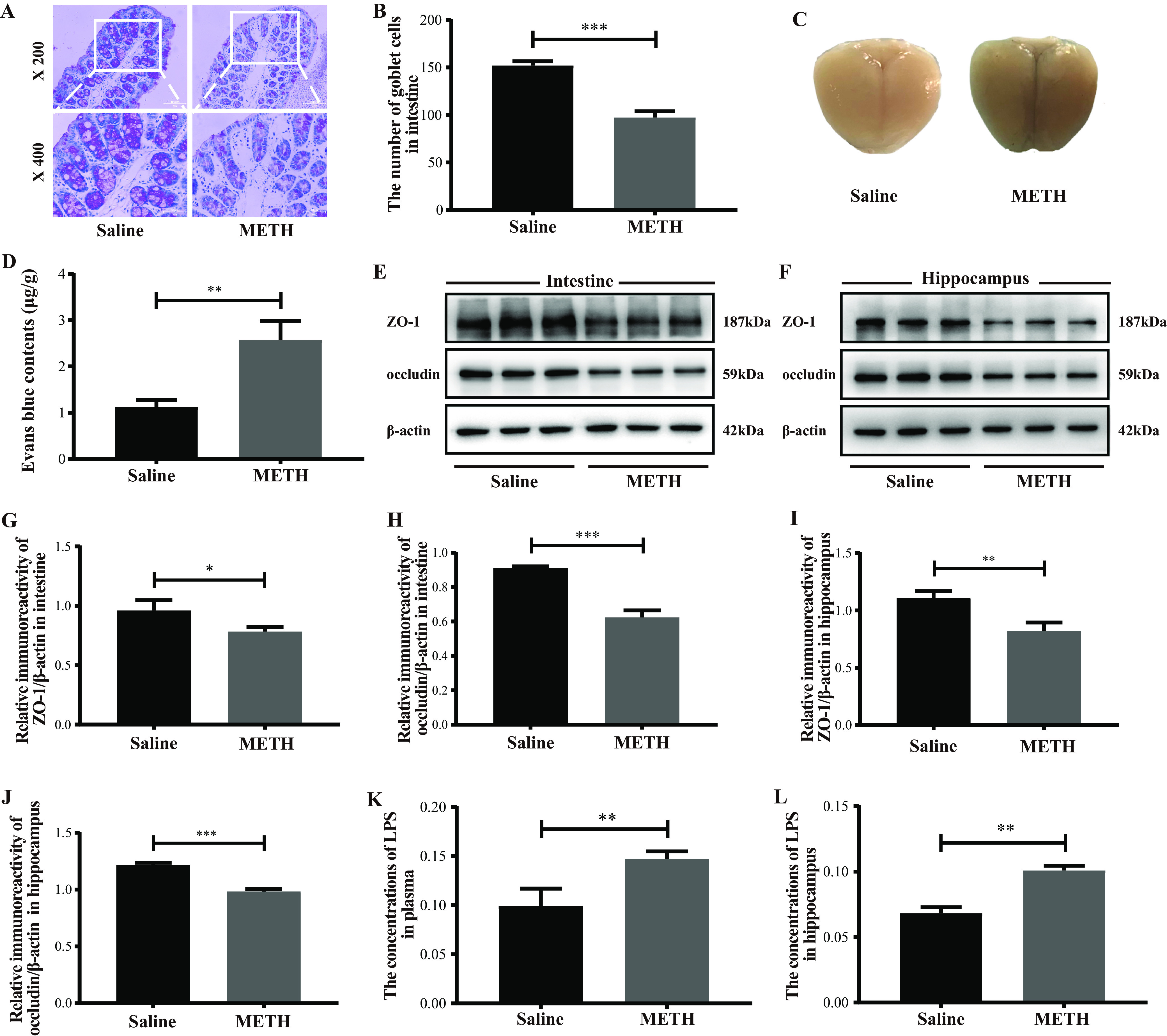
Chronic METH exposure disrupted the intestinal and BBB permeability and elevated LPS levels in the plasma and hippocampus. (A and B) Representative PAS staining (bars, 100 μm [×200 magnification] and 50 μm [×400 magnification]) and quantitative analyses of the number of intestine goblet cells of different groups. (C and D) Representative images and quantitative analyses of the Evans blue leakage in brains from different groups. (E and F) Representative protein immunoblot showing the expressions of tight junction proteins (ZO-1 and occludin) in the intestines and hippocampi of different groups. (G to J) Densities of immunoblot bands of ZO-1/β-actin and occludin/β-actin in the intestine and hippocampus. (K, L) Levels of LPS in the plasma and the hippocampus. Data are means and SEM (*n* = 10 to 12). *, *P* < 0.05; **, *P* < 0.01; ***, *P* < 0.001.

The permeability of the intestinal mucous barrier and BBB was evaluated by periodic acid-Schiff (PAS) staining and the Evans blue leakage test, respectively. The tight junction proteins (ZO-1 and occludin) expressed in the intestine and hippocampus were assessed by Western blotting. The levels of lipopolysaccharide (LPS) were detected with a *Limulus* amebocyte lysate assay. Our results showed that the number of intestinal goblet cells was notably decreased, while the contents of brain Evans blue were significantly greater in the METH group than in the saline group (Fig. 3A to D). Additionally, the intestinal and hippocampal ZO-1 and occludin expressions were significantly lower in the METH group than in the saline group (Fig. 3E to J). These results indicate that the intestinal and BBB permeability were disrupted after chronic METH exposure. As shown in Fig. 3K and L, the levels of LPS were dramatically higher in the plasma and hippocampi of the METH group than in the saline group. Together, these data indicated that chronic METH exposure might elevate the LPS contents in the plasma and hippocampus by increasing the permeability of the intestine and BBB, respectively.

### Changes of microglial M1/M2 phenotypes and the secreted factors in the hippocampus after chronic METH exposure.

**FIG 4:**
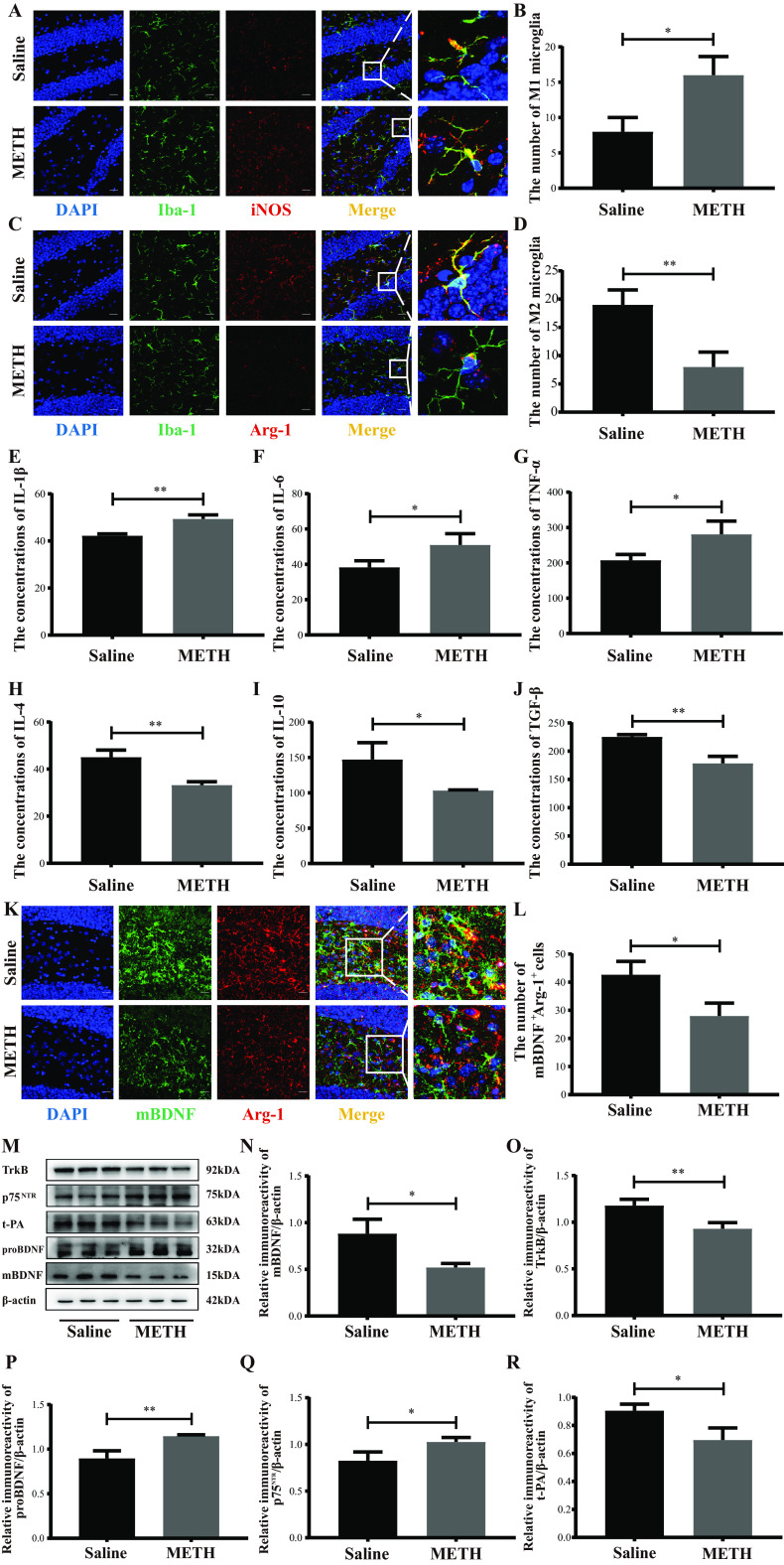
Changes in microglial M1/M2 phenotypes and the secreted factors in the hippocampus after chronic METH exposure. (A and C) Representative immunofluorescence staining images (magnification, ×400; bar = 20 μm) of nuclei (DAPI; blue), total microglia (Iba-1^+^; green), M1 microglia (iNOS^+^; red), and M2 microglia (Arg-1^+^; red) in the hippocampal dentate gyri of different groups. (B and D) Quantitative analysis of the number of Iba1^+^ iNOS^+^ cells (M1 microglia) and Iba1^+^ Arg-1^+^ cells (M2 microglia) in the hippocampal dentate gyri of different groups. (E to J) Levels of proinflammatory cytokines (IL-1β, IL-6, and TNF-α) and anti-inflammatory cytokines (IL-4, IL-10, and TGF-β) in the hippocampi of different groups. (K) Representative immunofluorescence staining images (magnification, ×400; bar = 20 μm) of nuclei (DAPI; blue), M2 microglia (Arg-1^+^; red), and mBDNF (mBDNF^+^; green) in the hippocampal dentate gyri of different groups. (L) Quantitative analysis of the number of Iba1^+^mBDNF^+^ cells in the hippocampal dentate gyri of different groups. (M) Representative protein immunoblots showing the expression of proteins regulating the BDNF signaling system (mBDNF, TrkB, proBDNF, p75^NTR^, and t-PA) of different groups. (N to R) Densities of immunoblot bands of mBDNF/β-actin, TrkB/β-actin, proBDNF/β-actin, p75^NTR^/β-actin, and t-PA/β-actin in the hippocampi of different groups. Data are means and SEM (*n* = 10 to 12). *, *P* < 0.05; **, *P* < 0.01.

Iba-1, inducible nitric oxide synthase (iNOS), and Arg-1 were used as indicators of total microglia, M1 microglia, and M2 microglia, respectively. The microglial M1/M2 phenotypes were detected by double immunofluorescence. The levels of their secreted proinflammation/anti-inflammation cytokines were assessed by enzyme-linked immunosorbent assay (ELISA). We found that the numbers of Iba-1^+^ iNOS^+^ cells (M1 microglia) were significantly increased while those of Iba-1^+^ Arg-1^+^ cells (M2 microglia) were considerably reduced in the hippocampal dentate gyri of the METH group compared to those in the saline group (Fig. 4A to D). Meanwhile, the concentrations of proinflammatory cytokines (IL-1β, IL-6, and TNF-α), which may be secreted by M1 microglia, were significantly elevated, with concomitantly decreased expression of anti-inflammatory cytokines (IL-4, IL-10, and TGF-β), which can be produced by M2 microglia, in the hippocampi of the METH group compared with the saline group (Fig. 4E to J). These results indicated that chronic METH exposure might facilitate M1 microglia activation while restraining M2 microglia activation in the hippocampus.

M2 microglia can also secrete BDNF, which is the most functionally diverse member of the neurotrophin (NT) family. Double immunofluorescence was used to assess mBDNF secreted by M2 microglia. The expressions of proteins regulating the BDNF signaling system were analyzed by Western blotting. Our results showed that the numbers of mBDNF^+^ Arg-1^+^ cells were significantly lower in the hippocampi of the METH group than those of the saline group (Fig. 4K and L). Meanwhile, the levels of mBDNF, its receptor TrkB, and t-PA, which cleaves the proBDNF to mBDNF, were significantly decreased, with concomitant increased expression of proBDNF and its receptor p75^NTR^, in the METH group compared with the saline group (Fig. 4M to R). These results indicated that chronic METH exposure might enhance proBDNF-p75^NTR^ signaling while suppressing mBDNF-TrkB signaling.

### Reduction of neurogenesis and synapse-associated proteins in the hippocampus after chronic METH exposure.

**FIG 5:**
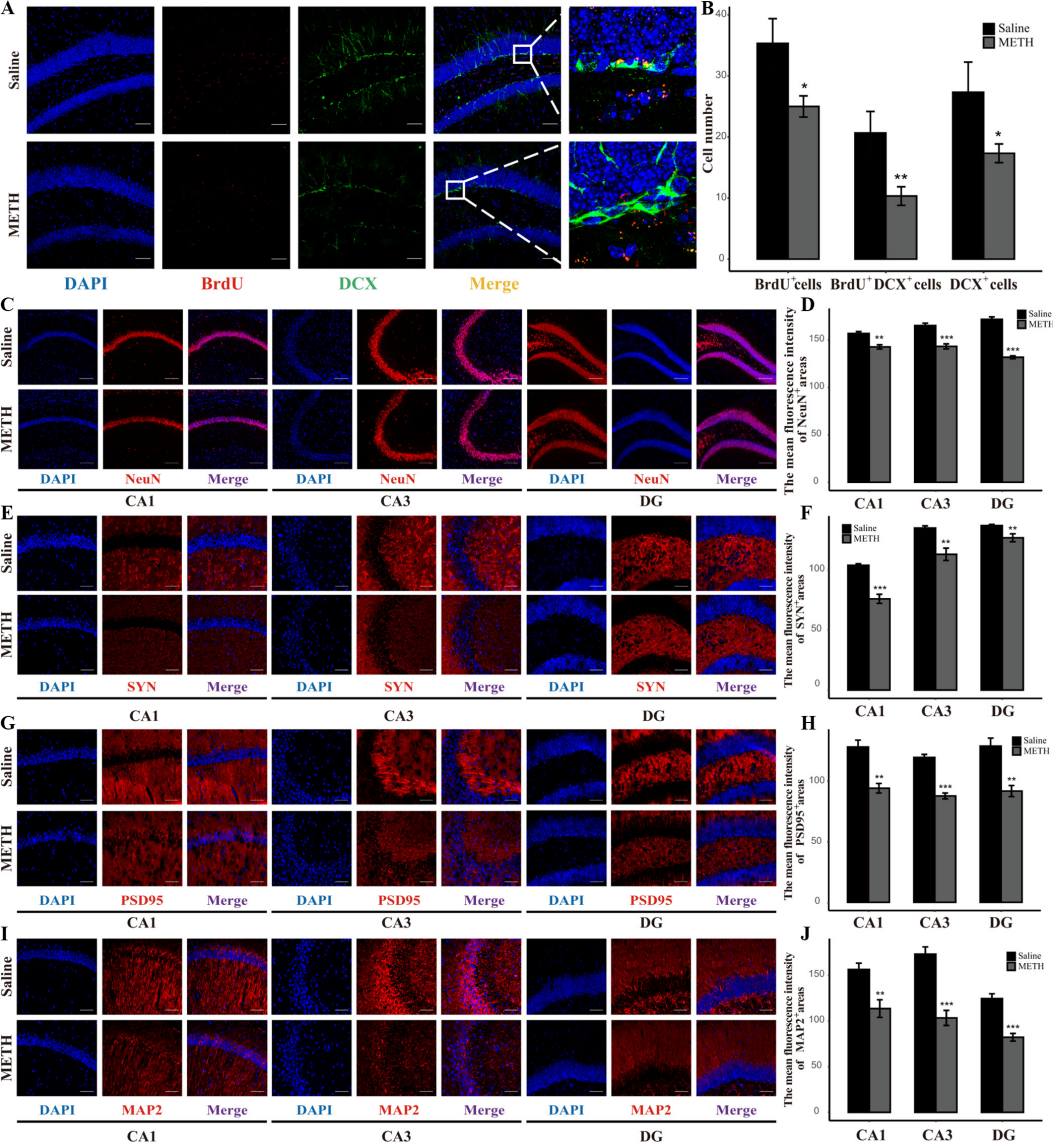
Reduction of neurogenesis and synapse-associated proteins in the hippocampus after chronic METH exposure. (A) Representative immunofluorescence staining images (magnification, ×200; bar = 50 μm) of nuclei (DAPI; blue), proliferating NSCs (BrdU^+^; red), and immature neuronal differentiation (DCX^+^; green) in the hippocampal dentate gyri of different groups. (B) Quantitative analysis of the number of BrdU^+^ cells (indicators of proliferating NSCs), DCX^+^ cells (indicators of immature neuronal differentiation), and BrdU^+^ DCX^+^ cells (indicators of immature neurons which differentiated from proliferating NSCs) in the hippocampal dentate gyri of different groups. (C) Representative immunofluorescence staining images (magnification, ×100; bar = 130 μm) of nuclei (DAPI; blue) and mature neurons (NeuN^+^; red) in the CA1, CA3, and DG regions from the hippocampi of different groups. (D) Quantitative analysis of the mean fluorescence intensity of NeuN^+^ areas (indicators of mature neurons) in the CA1, CA3, and DG regions from the hippocampi of different groups. (E, G, and I) Representative immunofluorescence staining images (magnification, ×200; bar = 60 μm) of nuclei (DAPI; blue) and synapse-associated proteins (SYN^+^, PSD95^+^, and MAP2^+^; red) in the CA1, CA3, and DG regions of the hippocampi of different groups. (F, H, and J) Quantitative analysis of the mean fluorescence intensity of SYN^+^, PSD95^+^, and MAP2^+^ areas in the CA1, CA3, and DG regions of the hippocampi of different groups. Data are means and SEM (*n* = 10 to 12). *, *P* < 0.05; **, *P* < 0.01; ***, *P* < 0.001.

Neurogenesis is a process by which neural stem cells (NSCs) proliferate, differentiate into immature neurons, and eventually form mature neurons. Neurogenesis continues throughout adulthood in the mammalian dentate gyrus (DG) of the hippocampus. BrdU (an indicator of proliferous NSCs), DCX (an indicator of immature neurons), and NeuN (an indicator of mature neurons) were stained by immunofluorescence. Our results showed that the BrdU^+^ cells, DCX^+^ cells, and BrdU^+^ DCX^+^ cells were significantly lower in the hippocampal dentate gyri of the METH group than the saline group (Fig. 5A and B). These results revealed that METH exposure restrained the proliferation and differentiation of NSCs. Meanwhile, the expression of NeuN was downregulated in the DG, Cornu ammonis 1 (CA1), and CA3 of the hippocampi of the METH group compared to those of the saline group (Fig. 5C and D), which indicated that chronic METH exposure inhibited the neuronal maturity in the hippocampus. Together, our data suggest that neurogenesis is suppressed after chronic METH exposure.

Moreover, three synapse-associated proteins that are vital for synaptic function were stained by immunofluorescence, including synaptophysin (SYN; an indicator of presynaptic plasticity which is located in the presynaptic membrane vesicle), PSD95 (an indicator of postsynaptic plasticity which is located in the postsynaptic membrane), and MAP2 (an indicator of synaptic plasticity which is situated in axons and dendrites of neurons). As shown in Fig. 5E to J, the expression levels of SYN, PSD95, and MAP2 were significantly lower in the hippocampi of the METH group than the saline group. These results indicate that chronic METH exposure might inhibit the synapse plasticity in the hippocampus.

### Microglial M1/M2 phenotypes mediated the relationship between specific taxa and spatial learning and memory ability after chronic METH exposure.

**FIG 6:**
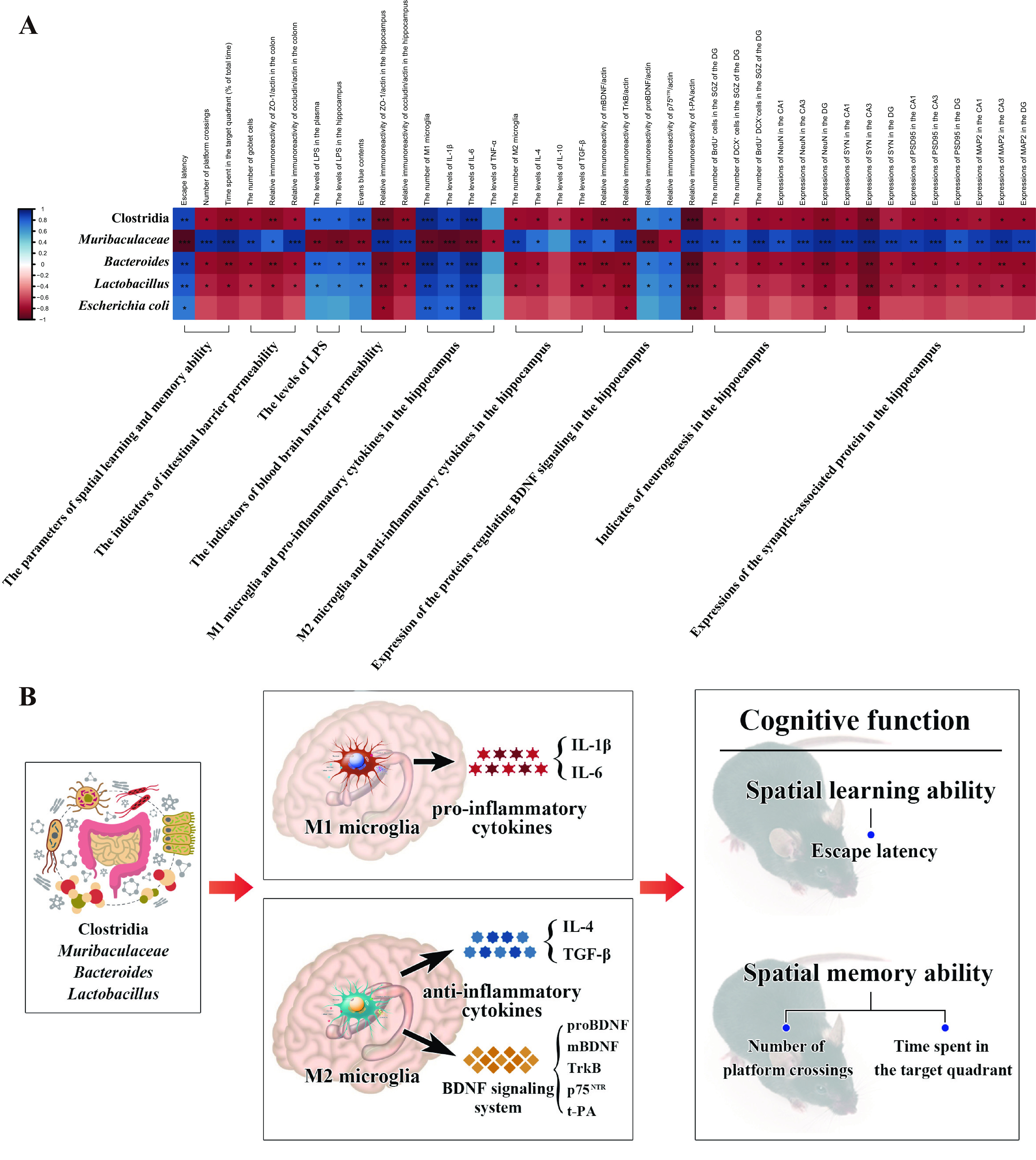
Microglial M1/M2 phenotypes mediate the relationship between specific taxa and spatial learning and memory ability after chronic METH exposure. (A) Correlation between the relative abundance of discriminant taxa and serial biochemical and behavioral indicators of chronically METH-exposed mice. (B) Microglial M1/M2 phenotypes and their secreted factors mediate the effects of specific taxa on spatial learning and memory after chronic METH exposure.

Figure 6A shows the association of the discriminant taxa with serial biochemical and behavioral indicators. Notably, the relative abundance of *Clostridia*, *Bacteroides*, and *Lactobacillus* showed positive correlations with plasma and hippocampal levels of LPS, M1 microglia, proinflammatory cytokines (IL-1β and IL-6), proBDNF, and p75^NTR^ while simultaneously being negatively associated with M2 microglia, levels of anti-inflammatory cytokine (IL-4 and TGF-β), mBDNF, TrkB, and t-PA, and spatial learning and memory ability. Conversely, these indicators of the above-mentioned molecular biology and spatial learning and memory ability showed an opposite correlation with the relative abundance of *Muribaculaceae*.

To further address whether microglial M1/M2 phenotypes have mediating roles and assess the mediation proportion in the effect of specific taxa (*Clostridia*, *Bacteroides*, *Lactobacillus*, and *Muribaculaceae*) on spatial learning and memory ability, mediation analyses were conducted with indicators of microglial M1/M2 phenotypes and their respectively secreted factors as the mediators and scores of the MWM as the outcome (30, 31). We conducted mediation analyses only when the mediators and the outcome were simultaneously correlated with the specific taxon. The results provided average causal mediation effects (ACMEs), i.e., the association between a particular taxon and MWM scores through indicators of microglial M1/M2 phenotypes and their respectively secreted factors, and provided total effect and mediation proportion. Generally, we observed serial significant (*P* value for ACME < 0.05) mediation effects of microglial M1/M2 phenotypes and their secreting factors between the selected taxa (*Clostridia*, *Bacteroides*, *Lactobacillus*, and *Muribaculaceae*) and spatial learning and memory ability (Fig. 6B; Tables S1 to S4). For example, the effect of *Muribaculaceae* via M1 microglia on spatial learning and memory ability was significant, with the mediation effect through M1 microglia accounting for 53.4% of escape latency (an indicator of spatial learning ability), 55.7% of the number of platforms crossing (indicator of spatial memory ability), and 54.6% of time spent in the target quadrant (indicator of spatial memory ability). Stronger ACMEs through the proinflammatory cytokines (such as IL-1β and IL-6) on spatial learning and memory ability also exist. Meanwhile, we also observed mediation effects through M2 microglia and their secreted factors on spatial learning and memory ability. Together, these mediation analysis results clearly validated our 16S rRNA, molecular, morphological, and behavioral results, which indicated that *Clostridia*, *Bacteroides*, *Lactobacillus*, and *Muribaculaceae* might contribute to the deterioration of spatial learning and memory through regulating of microglial M1/M2 phenotypes after chronic METH exposure.

### Fecal microbiota transplantation attenuated chronic-METH-exposure-associated spatial learning and memory impairment by reviving microglial M1/M2 phenotype homeostasis.

**FIG 7:**
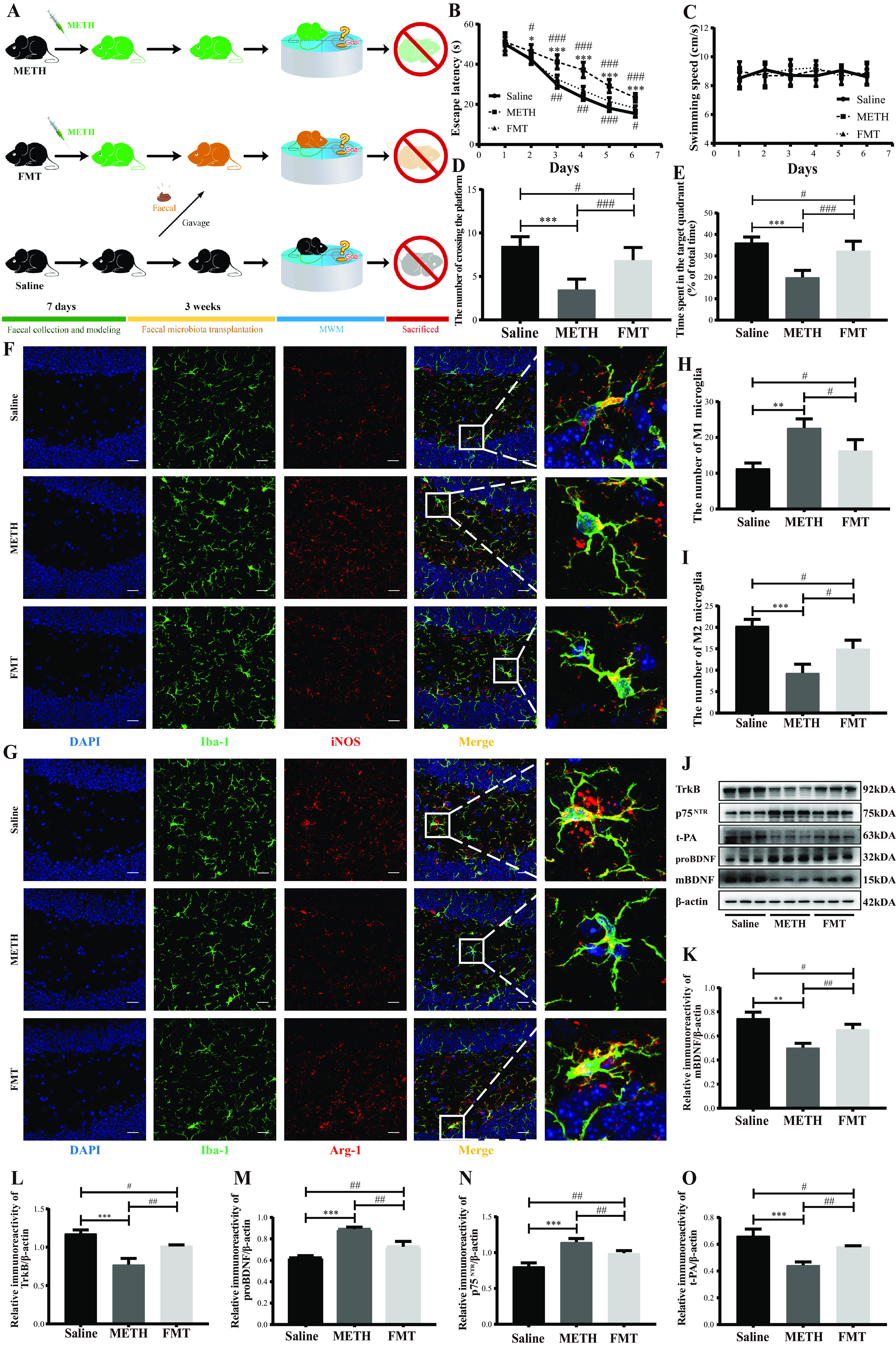
Fecal microbiota transplantation attenuated chronic-METH-exposure-induced spatial learning and memory impairment by reviving microglial M1/M2 phenotype homeostasis. (A) Experimental design. Chronically METH-exposed mice were colonized with fecal samples from the mice in the saline group. (B and C) Escape latency and the swimming speed during the place navigation of the MWM test showed that spatial learning ability was attenuated by fecal microbiota transplantation from donor mice. (D and E) Numbers of instances of crossing the platform from a previously determined spot and time spent in the target quadrant (percent of total time) during the spatial probe of the MWM test indicated that spatial memory ability was attenuated by fecal microbiota transplantation from the donor mice. (F and G) Representative immunofluorescence staining images (magnification, ×400; bar = 20 μm) of nuclei (DAPI; blue), total microglia (Iba-1^+^; green), M1 microglia (iNOS^+^; red), and M2 microglia (Arg-1^+^; red) in the hippocampal dentate gyri of different groups. (H and I) Quantitative analysis of the number of Iba1^+^ iNOS^+^ cells (M1 microglia) and Iba1^+^ Arg-1^+^ cells (M2 microglia) in the hippocampal dentate gyri of different groups. (J) Representative protein immunoblots showing the expressions of proteins regulating the BDNF signaling system (mBDNF, TrkB, proBDNF, p75^NTR^, and t-PA) of different groups. (K to O) Densities of immunoblot bands of mBDNF/β-actin, TrkB/β-actin, proBDNF/β-actin, p75^NTR^/β-actin, and t-PA/β-actin in the hippocampi of different groups. Data are means and SEM (*n* = 12 to 14). *, *P* < 0.05, **, *P* < 0.01, and ***, *P* < 0.001, compared with the saline group; #, *P* < 0.05, ##, *P* < 0.01, and ###, *P* < 0.001, compared with the FMT group.

Fecal samples from the control mice were selected and transplanted into the chronically METH-exposed mice (Fig. 7A). Spatial learning and memory abilities were detected by MWM. With the increase of training days in the place navigation test (days 1 to 6), all groups gradually reduced the escape latency required to look for the hidden platform (Fig. 7B). There was a significant interaction between groups and times [*F*(10, 165) = 10.398, *P* < 0.001] with regard to the escape latency. *Post hoc* analyses showed that the escape latency was considerably shorter in the fecal microbiota transplantation (FMT) and control groups than in the METH group from the 2nd day to the 6th day, and the statistical evaluation showed more significance in the control group than the METH group from the 3rd day to the 6th day. For swimming speed, no significant differences were observed between groups as the training days progressed (Fig. 7C). The spatial probe trial on the 7th day showed that the FMT and control groups crossed the platform more often and spent a longer time in the target quadrant than the METH group, with remarkable alteration in the control group (Fig. 7D and E). These results indicated that fecal microbiota transplantation from control donors could partially remedy spatial learning and memory impairment after chronic METH exposure.

To explore the underlying mechanism of neuroprotective effects induced by fecal microbiota transplantation, we investigated microglial M1/M2 phenotypes by double immunofluorescence. The numbers of Iba-1^+^ iNOS^+^ cells (M1 microglia) were significantly reduced while those of the Iba-1^+^ Arg-1^+^ cells (M2 microglia) were dramatically elevated in the FMT and control groups compared to the METH group and exhibited a more marked difference in the control group (Fig. 7F to I). The results showed that FMT might partially restore the microglial M1/M2 phenotype homeostasis by switching M1 to M2 microglia.

To further clarify whether fecal microbiota transplantation-induced transformation of microglial M1/M2 phenotypes could subsequently cause alteration of the BDNF signaling system, we evaluated the expression of proteins regulating the BDNF signaling system by Western blotting. Our results showed that the levels of mBDNF, TrkB, and t-PA were consistently upregulated whereas the expression of proBDNF and p75^NTR^ was simultaneously downregulated in the FMT and control groups compared with the METH group, with more remarkable changes in the control group (Fig. 7J to O). These results demonstrated that fecal microbiota transplantation might enhance mBDNF-TrkB signaling and restrain proBDNF-p75^NTR^ signaling in the hippocampi of chronically METH-exposed mice.

Overall, our data indicated that chronic-METH-exposure-associated spatial learning and memory impairment might be mitigated by fecal microbiota transplantation with resulting regulation of microglial M1/M2 phenotype status and the subsequent switching of proBDNF-p75^NTR^ to mBDNF-TrkB signaling.

## DISCUSSION

The present study reports that the gut microbiota is critically involved in spatial learning and memory impairment after chronic METH exposure. Our work revealed that METH exposure-induced gut microbiota perturbation triggers microglial M1/M2 phenotype transformation and the subsequent shift of proBDNF-p75^NTR^/mBDNF-TrkB signaling, which induces suppression of hippocampal neurogenesis and synaptic plasticity, thereby contributing to spatial learning and memory deterioration. Specifically, we identified that specific gut microbiota taxa (*Clostridia*, *Bacteroides*, *Lactobacillus*, and *Muribaculaceae*) might dramatically affect the homeostasis of microglial M1/M2 phenotypes and eventually contribute to spatial learning and memory decline after chronic METH exposure (Fig. 6). Finally, we found fecal microbial transplantation could alleviate spatial learning and memory deficits by restoring homeostasis of hippocampal microglial M1/M2 phenotypes and proBDNF-p75^NTR^/mBDNF-TrkB signaling. Our work comprehensively integrated the gut microbiota, brain immune cells (microglia) and their secreted factors, subsequent hippocampal neural responses, and the consequent effects on cognition of chronically METH-exposed mammals. To our best knowledge, this is the first study focusing on the role and cellular and molecular mechanism of the microbiota on chronic-METH-associated cognitive deterioration and promoting potential nonpharmacological treatment by targeting the gut microbiota to restore spatial learning and memory deficits after chronic METH exposure.

In our research, we adopted a chronic-METH-exposure model, which reliably induces spatial learning and memory impairment in mice (32, 33). As expected, we found that spatial learning and memory were deteriorated after the chronic METH exposure. Meanwhile, we analyzed the influence of chronic METH exposure on the intestinal microbiota. Burgeoning evidence shows that gut bacterial dysbiosis occurs after addictive drug applications, including heroin, opioids, morphine, and methamphetamine (34). However, due to the different types, dosages, and duration of addictive drugs, the alterations of the intestinal microbiota are inconsistent. In the present study, we found that opportunistically pathogenic bacteria, including *Clostridia*, *Lachnospiraceae*, *Bacteroides*, and Escherichia coli, which have proinflammatory effects, were the specific taxa enriched in the intestinal microbiotas of the chronically METH-exposed mice. It is reported that some particular taxa belonging to *Clostridia* are associated with the elevation of inflammation-related genes, including those encoding TNF-α, IL-1β, and IL-6 (35, 36). In rodents, *Lachnospiraceae* has frequently been found to be involved in cognitive dysfunction disease induced by type 1 diabetes, lead, and hepatic encephalopathy (37–39). Recent studies investigated the increased abundance of Gram-negative taxa such as Escherichia coli and *Bacteroides* in AD patients and cognition-impaired elderly persons with brain amyloidosis (40, 41). Gram-negative bacteria heavily display LPS, which can leak from the destroyed intestinal barrier to the host’s circulatory system and cause neuroinflammation (42, 43), which is considered a crucial pathogenesis mechanism of AD (44–46). Unexpectedly, we found that probiotic *Lactobacillus* and Lactobacillus johnsonii were also distinct taxa enriched in the chronically METH-exposed mice. Similarly, several previous studies found that the abundance of *Lactobacillus* increased in the gut of AD patients (47, 48). The possible reason might be that changes in the gut microenvironment promote an increase in these particular probiotic bacterial strains but still fail to counteract the detrimental effects of the conditionally pathogenic bacteria on cognition. Moreover, our data showed that the *Muribaculaceae*, whose members produce SCFAs, is the representative taxon in the saline group. SCFAs are the most crucial microbial products with anti-inflammatory potential and maintain the gastrointestinal system’s homeostasis (49, 50). Decreasing SCFAs could deteriorate the gut permeability (51), making it possible for the detrimental microbiota products such as LPS to leak into the circulatory system and accelerate the progression of neurodegeneration diseases (52, 53). Furthermore, we found that spatial learning and memory ability are negatively related to the METH group-enriched taxa (*Clostridia*, *Bacteroides*, and *Lactobacillus*) but positively associated with the saline group-enriched taxon (*Muribaculaceae*) (Fig. 6A). This suggests that these taxa might be involved in the deterioration of spatial learning and memory after chronic METH exposure.

METH exposure deteriorates tight junction proteins, thus elevating the permeability of the gut and the BBB (11, 54), making it possible for bacterial-origin antigens, such as LPS, to enter the central nervous system (CNS). Earlier clinical research reported substantial LPS in the cardiovascular systems of deceased METH addicts (55). However, the trigger factors of the elevated endogenous LPS after METH exposure and its effect on neurofunction remain elusive. Our results showed that the plasma and hippocampal LPS concentrations were elevated in parallel with the disruption of the gut and BBB permeability after chronic METH exposure. Moreover, the plasma and hippocampal LPS levels were positively correlated with *Clostridia*, *Bacteroides*, and *Lactobacillus*, whereas they were negatively associated with *Muribaculaceae*. Interestingly, the correlations of these taxa and LPS are the opposite of their association with spatial learning and memory ability (Fig. 6A). Previous studies revealed that exogenous exposure to LPS potentiates METH-induced neurotoxicity (56, 57). Our results strongly indicate that specific taxa (*Clostridia*, *Bacteroides*, *Lactobacillus*, and *Muribaculaceae*) might contribute to the alteration of endogenous LPS after chronic METH exposure and, consequently, to the deterioration of spatial learning and memory.

LPS significantly activates the proinflammatory functional phenotype of microglia (M1) in a dose-dependent manner (58). Increasing evidence reveals that neuroinflammation exhibited by microglia plays a crucial role in METH-relevant neurotoxicity (23, 24). However, several published studies presented conflicting conclusions on the trigger factors of activated microglia after METH exposure. In *in vivo* studies, METH itself was primarily considered to activate the proinflammatory phenotype of microglia and induce secretion of proinflammatory cytokines in the CNS (59–61). However, a recent study demonstrated that proinflammatory cytokines underwent insignificant changes in METH-treated microglial cultures (62). The discordant results of *in vivo* and *in vitro* experiments indicated that other cerebral factors or neuronal danger-associated molecular patterns (DAMPs) rather than METH might mediate microglial activation *in vivo*. In the present study, we found that M1 microglia and proinflammatory cytokines (IL-1β, IL-6, and TNF-α) were augmented in the hippocampus after chronic METH exposure, which confirms previous studies (63–66). Moreover, we found that along with the overactivation of M1 microglia, M2 microglia were suppressed in the hippocampus after chronic METH exposure. Microglia have a phenotype-dependent dual effect on neurological function depending on the cerebral microenvironment (22). M2 microglia trigger the release of anti-inflammatory cytokines (e.g., IL-4, IL-10, and TGF-β) and neurotrophic factors such as BDNF to mitigate and repair injury caused by proinflammatory responses (67). Our data showed that M1 microglia and proinflammatory cytokines (IL-1β and IL-6) were positively correlated with *Clostridia*, *Bacteroides*, and *Lactobacillus* and negatively correlated with *Muribaculaceae*. Simultaneously, each of these particular taxa showed reverse correlations with M2 microglia and anti-inflammatory cytokines (IL-4 and TGF-β) (Fig. 6A). Recent evidence revealed that the intestinal microbiota and bacterium-derived macromolecules strongly impact microglial abundance, maturation, and activation under physiologic conditions (14, 15, 68). Our data strongly suggest the conceivable engagement of specific taxa (*Clostridia*, *Bacteroides*, *Lactobacillus*, and *Muribaculaceae*) in disturbance of microglial homeostasis by facilitating M1 and restraining M2 microglia under the pathophysiological conditions of chronic METH exposure. To the best of our knowledge, our study is the first to demonstrate such an association.

Solid evidence has shown that microglia regulate hippocampal neurogenesis (69) and promote synapse plasticity, on which learning is dependent, through M2 microglia-produced BDNF (25). Matsuzaki et al. reported that METH-induced neuronal death could be alleviated by modulation of BDNF (70). Two forms of BDNF exist in the CNS: proBDNF and mBDNF, which exert opposite neurofunctional regulation by separately binding to their high-affinity receptors p75^NTR^ and TrkB (71, 72). In our previous clinical study, we found that downregulation of the ratio of mBDNF to proBDNF was associated with cognitive impairment after METH exposure (26). However, the mechanism underlying the METH exposure-associated change of mBDNF/proBDNF remains unclear. In the current study, we found that M2 microglia-produced mBDNF was suppressed after chronic METH exposure. Meanwhile, the expression of TrkB, which specifically binds to mBDNF, and t-PA, which proteolytically cleaves proBDNF to mBDNF (73), was downregulated while the levels of proBDNF and p75^NTR^ were elevated in the hippocampi of chronically METH-exposed mice. Remarkably, we found that *Muribaculaceae* was positively correlated with M2 microglia, mBDNF, TrkB, and t-PA but negatively associated with proBDNF and p75^NTR^, while *Clostridia*, *Bacteroides*, and *Lactobacillus* exhibited the opposite correlations with M2 microglia and the proteins mentioned above (Fig. 6A). These associations indicated that these specific taxa might be involved in the shift of mBDNF-TrkB to proBDNF-p75^NTR^ signaling after chronic METH exposure. mBDNF promotes neurogenesis and facilitates synaptic plasticity, whereas proBDNF triggers proapoptotic and synaptic withdrawal (74–78). Thus, we further examined the neurological response to the specific taxon-associated alteration of the BDNF signaling system after chronic METH exposure. As expected, we found that neurogenesis and synaptic plasticity were inhibited in the hippocampi of chronically METH-exposed mice. Emerging evidence has indicated that METH exposure-induced reductions in newly generated neurons and synaptic plasticity may impair hippocampus-dependent learning and memory (79, 80). Thus, the present study supports a potential link between particular intestinal bacterial taxa, M2 microglia, the BDNF signaling system, and spatial learning and memory impairment after chronic METH exposure. Although the underlying mechanism of this model has not been precisely identified, our results add new evidence to support the idea that the intestinal microbiota can modify the metabolism of neurotransmitters (81) and neurotrophins (82) and alter the expression of molecules in the cerebral nervous system (83, 84).

Together, our research shows that specific taxa have significant correlation with serial indicators, including microglial M1/M2 phenotypes, proinflammatory/anti-inflammatory cytokines, proBDNF-p75^NTR^/mBDNF-TrkB signaling, neurogenesis, synaptic plasticity, and spatial learning and memory ability (Fig. 6A). Considering that the results of correlation analyses cannot simultaneously reveal the directional relationships of multiple factors, to further examine whether and how much microglial M1/M2 phenotypes contribute to the effect of these specific taxa on spatial learning and memory, mediation analyses were conducted with significantly correlated indicators of M1 or M2 microglia and their secreted factors as the mediators and scores for spatial learning and memory as the outcome. The mediation analysis identified a unified model incorporating particular taxa (*Clostridia*, *Bacteroides*, *Lactobacillus*, and *Muribaculaceae*), microglial M1/M2 phenotypes, and spatial learning and memory (Fig. 6B). Although the detailed mechanism needs to be further elucidated by distinct bacterial strain culture experiments, our mediation analysis results confirm and add evidence that these specific taxa might determine the state of microglial M1/M2 phenotypes and are therefore essential for deterioration of spatial learning and memory after chronic METH exposure. These data appear to imply that there are synergistic effects of interventions with these specific taxa and microglial M1/M2 phenotypes to alleviate spatial learning and memory deficits after chronic METH exposure.

Finally, we sought to assess whether fecal microbiota transplantation would restore spatial learning and memory impairment via regulating microglial M1/M2 phenotypes and the subsequent change of the BDNF signaling system in the hippocampi of chronically METH-exposed mice. Fecal microbiota transplantation has become a promising therapeutic approach for GI tract-related disorders and CNS diseases (85, 86). In the current study, we found that FMT from control donors led to improvement of spatial learning and memory, paralleled by alteration of M1 to M2 microglia and the subsequent switch of proBDNF-p75^NTR^ to mBDNF-TrkB signaling in the hippocampi of chronically METH-exposed mice. These findings provided further validation that intestinal taxa might be trigger factors of spatial learning and memory deterioration by regulating hippocampal microglial M1/M2 phenotypes status. Our work highlights fecal microbiota transplantation as a potential therapeutic approach for cognitive dysfunction after chronic METH exposure.

In conclusion, our findings strongly support the critical role of specific gut microbiota taxa (*Clostridia*, *Bacteroides*, *Lactobacillus*, and *Muribaculaceae*) in chronic-METH-exposure-associated spatial learning and memory deficits by regulating the transformation of microglial M1/M2 phenotypes and subsequently influencing the neuroactive compounds (including neuroinflammation cytokines and molecules of the BDNF signaling system), thus promoting degeneration of the hippocampal neural process. It is noteworthy that this work provides comprehensive and new insights into the roles of particular gut microbiota taxa in spatial learning and memory deficits through disturbance of microglial M1/M2 phenotype homeostasis and promotes the therapeutic possibilities of modifications targeting gut microbiota taxa for neurodegeneration after chronic METH exposure.

## MATERIALS AND METHODS

### Mouse model and treatment.

Animal experiments were performed in accordance with the ethical standards of the Binzhou Medical University ethical committee. C57BL/6 mice (male, 7 weeks old, weighing 25 ± 2 g) were purchased from the Jinan Pengyue Laboratory Animal Breeding Company (Jinan, Shandong, China). All mice were housed (4 per cage) under standardized conditions (23 ± 2°C, 50% ± 5% humidity) and a 12-h light/dark cycle with free access to chow and water. After 7 days of adaptive feeding, the mice were randomly divided into the METH and saline groups. The mice in the METH group were injected intraperitoneally with 10 mg of METH/kg of body weight, and the mice in the saline group were injected intraperitoneally with a parallel regimen of saline given at the volume (400 μL) used for their METH-treated counterparts. The injection procedure was implemented once daily for seven consecutive days and carried out at a fixed time.

### Spatial learning and memory.

The MWM was used to evaluate spatial learning and memory. The protocol of the MWM was described in our previous study (87).

### Determination of biochemical parameters of plasma and tissues.

Blood from anesthetized mice was drawn into endotoxin-free centrifuge tubes containing heparin sodium by orbital puncture, and plasma was obtained by centrifuging the blood at 3,000 × *g* for 2 min at 4°C. Afterward, the separated hippocampus was homogenized with sterile phosphate-buffered saline (PBS), and the supernatants were collected after centrifugation at 5,000 × *g* for 10 min at 4°C. The prepared plasma and hippocampal supernatants were detected with a chromogenic matrix endotoxin quantitation kit (Xiamen Bioendo Technology Co., Ltd., China) to evaluate the levels of LPS, and the protocols were carried out in strict accordance with the kit instructions. The hippocampal IL-6, IL-1β, TNF-α, IL-4, IL-10, and TGF-β levels were detected by mouse ELISA kits (Enzyme-linked Biotechnology, Dalian, China) according to the manufacturer’s instructions.

### Microbial analysis.

Fresh fecal samples were collected and stored at −80°C. Total genome DNA from samples was extracted using the cetyltrimethylammonium bromide (CTAB) method. The V4 region of the 16S rRNA genes were amplified using a specific primer (515F, GTGYCAGCMGCCGCGGTAA; 806R, GGACTACHVGGGTWTCTAAT) with the barcode. All PCRs were carried out with Phusion high-fidelity PCR master mix (New England Biolabs, Ipswich, MA, USA). After extraction, the PCR product was purified, and the library was generated and sequenced on an Illumina NovaSeq 6000 platform. Alpha diversity was calculated with QIIME (version 1.7.0). Beta diversity on weighted UniFrac distance was calculated by QIIME software (version 1.9.1), and analysis of similarity (ANOSIM) and ADONIS tests were performed to estimate statistical differences between the two groups. The linear discriminant analysis effect size was measured by LEfSe software (version 1.0), and the LDA value was set to 4. The evolutionary tree of characteristic taxa was generated by IQtree software (version 2.1.2; https://github.com/iqtree/iqtree2/releases/tag/v2.1.2) as the tree-building method and then illustrated by iTOL (version 6.3.2; https://itol.embl.de/).

### Metabolomics analysis.

Fecal samples were collected, and supernatants were extracted. Ultrahigh-performance liquid chromatography–tandem mass spectrometry (UHPLC-MS/MS) analysis was performed with a Vanquish UHPLC system (Thermo Fisher, Germany) coupled with an Orbitrap Q Exactive HF mass spectrometer (Thermo Fisher, Germany). The samples were injected onto a Hypersil Gold column (100 by 2.1 mm, 1.9 μm) using a 17-min linear gradient at a flow rate of 0.2 mL/min. The eluents for the positive polarity mode were eluent A (0.1% formic acid in water) and eluent B (methanol). The eluents for the negative polarity mode were eluent A (5 mM ammonium acetate, pH 9.0) and eluent B (methanol). The solvent gradient was set as follows: 2% B, 1.5 min; 2 to 85% B, 3 min; 100% B, 10 min; 100 to 2% B, 10.1 min; 2% B, 12 min. A Q Exactive HF mass spectrometer was operated in positive/negative polarity mode with a spray voltage of 3.5 kV, capillary temperature of 320°C, sheath gas flow rate of 35 lb/in^2^, auxiliary gas flow rate of 10 L/min, lens RF level of 60, and auxiliary gas heater temperature of 350°C.

### Intestinal permeability.

The intestinal permeability was determined by quantifying mucus-containing goblet cells and the level of the tight junction proteins (ZO-1 and occludin) expressed in the intestine. The quantity of mucus-containing goblet cells was detected by periodic acid-Schiff staining. The isolated intestine was dehydrated in a graded ethanol series, cleared in xylene, and embedded in paraffin. The intestinal paraffin blocks were cut into sections 4 μm thick with a paraffin-slicing microtome and then stained with glycogen periodic acid-Schiff (PAS/hematoxylin) stain kit (Solarbio, Beijing, China). Goblet cells were observed by optical microscopy and counted by using Image J software. The tight junction proteins (ZO-1 and occludin) expressed in the intestine were assessed by Western blotting.

### Blood-brain barrier permeability.

Blood-brain barrier permeability was checked by the Evans blue leakage test. Mice were administered 2% Evans blue (Sigma, St. Louis, MO, USA) at a dosage of 4 mL/kg by tail vein injection. After 4 h, anesthetized mice were perfused with PBS. The isolated brains were homogenized with formamide and placed in a water bath at 60°C for 24 h. Then, the supernatants were collected after centrifugation at 4,000 × *g* for 20 min. The optical density (OD) values were measured at a wavelength of 620 nm with a microplate reader.

### Immunofluorescence.

Mice were injected intraperitoneally with 50 mg/kg BrdU (Sigma, St. Louis, MO, USA), once daily for five consecutive days. Afterward, isolated brains were dehydrated in 30% sucrose followed by embedding in optimal cutting temperature compound (OCT) and then cut into frozen slices. After washing, the slices were blocked with 10% goat serum (ZSGB-BIO, Beijing, China). For BrdU immunofluorescence staining, before blocking, slices were incubated with 1 N HCl for DNA denaturation and neutralized with 0.1 M sodium borate buffer (pH 8.5). The slices were incubated at 4°C overnight with primary antibodies: rabbit anti-Iba-1 (Wako, Osaka, Japan), mouse anti-iNOS (Santa Cruz Biotechnology, Santa Cruz, CA, USA), mouse anti-Arg-1 (Santa Cruz Biotechnology, Santa Cruz, CA, USA), rabbit anti-mBDNF (Abcam, Cambridge, UK), rabbit anti-DCX (Abcam, Cambridge, UK), mouse anti-BrdU (Abcam, Cambridge, UK), rabbit anti-NeuN (Cell Signaling Technology, Danvers, MA, USA), rabbit anti-synaptophysin (SYN) (Proteintech, Wuhan, China), rabbit anti-PSD95 (Cell Signaling Technology, Danvers, MA, USA), and rabbit anti-MAP2 (Cell Signaling Technology, Danvers, MA, USA). After returning to room temperature the next day, the slices were washed and then incubated with respective fluorescent-labeled secondary antibodies (Abbkine, Redlands, CA, USA). 4′,6-Diamidino-2-phenylindole (DAPI; Solarbio Technology, Beijing, China) was used to stain the cell nucleus. The numbers of Iba-1^+^ iNOS^+^ cells (M1 microglia), Iba-1^+^ Arg-1^+^ cells (M2 microglia), mBDNF^+^ Arg-1^+^ cells, BrdU^+^ cells, DCX^+^ cells, and BrdU^+^ DCX^+^ cells were obtained by confocal laser scanning microscopy. The fluorescence intensity of NeuN, SYN, PSD95, and MAP2 was observed by fluorescence microscopy. The images were analyzed by ImageJ software.

### Protein extraction and Western blotting.

The hippocampus and intestine were homogenized in lysis buffer containing phosphatase and protease inhibitors (Solarbio, Beijing, China). The tissue supernatant concentrations were quantified using a bicinchoninic acid (BCA) protein concentration assay kit (Beyotime, Shanghai, China). Proteins were loaded into the wells of 10% or 12% SDS-PAGE gels and then were transferred onto polyvinylidene difluoride (PVDF) membranes. The membranes were blocked with 5% skim milk at room temperature for 2 h and then incubated at 4°C overnight with primary antibodies: rabbit anti-occludin (Proteintech, Wuhan, China), rabbit anti-ZO-1 (Abcam, Cambridge, UK), rabbit anti-mBDNF (Abcam, Cambridge, UK), rabbit anti-TrkB (Abcam, Cambridge, UK), mouse anti-proBDNF (Santa Cruz Biotechnology, Santa Cruz, CA, USA), rabbit anti-p75^NTR^ (Abcam, Cambridge, UK), rabbit anti-t-PA (Abcam, Cambridge, UK), and mouse anti-β-actin (Proteintech, Wuhan, China). The next day, the membranes were washed with Tris-buffered saline with Tween 20 (TBST). Then, the membranes were incubated at room temperature for 1 h with the appropriate horseradish peroxidase (HRP)-conjugated secondary antibodies (Proteintech, Wuhan, China). After being washed with TBST, the protein bands were treated with a superenhanced chemiluminescence kit (Novland, Shanghai, China), and images were captured with a chemiluminescence imager. The band densities were analyzed by ImageJ software.

### Fecal microbiota transplantation.

The mice were randomly divided into the control group, the METH group, and the FMT group. The fecal samples from the control group were prepared as described in a previous study (88). The mice in the METH group and the FMT group were injected intraperitoneally with METH for 7 days, as described for the chronic-METH-exposure experiment. The fecal samples from the control group were resuspended in sterile PBS (0.1 g/mL). After vigorous vortex mixing, the supernatants were obtained by centrifugation at 68 × *g* for 2 min (Eppendorf, Germany). Then, 300-μL supernatants were administered to the mice in the FMT group by gavage once per day at a fixed time for 21 consecutive days.

### Statistical analysis.

Statistical analysis was performed using SPSS software (version 22.0; IBM, Chicago, IL, USA) and R software (version 4.2.1; R Core Team, Boston, MA, USA). Data are presented as means and standard errors of the means (SEM). The normality of the data distribution was evaluated by the Shapiro-Wilk test. Data with a normal distribution were analyzed using a two-tailed Student’s *t* test to compare two groups. One-way analysis of variance (ANOVA) was followed by Fisher’s least-significant-difference (LSD) *post hoc* test to compare three groups. Escape latency and swimming speed were analyzed by two-way repeated-measures ANOVA with the Bonferroni *post hoc* test. As appropriate, correlations between specific taxa, behavioral parameters, and molecular biological indexes were assessed by Pearson or Spearman correlation analysis. Mediation analyses were performed with the R package mediation version 4.5.0. *P* values of <0.05 were considered statistically significant.

### Data availability.

The 16S rRNA gene sequencing data are available at NCBI’s Sequence Read Archive (SRA) database under BioProject accession number PRJNA926793. The metabolomic data have been deposited in the EMBL-EBI MetaboLights database (https://www.ebi.ac.uk/metabolights) with the identifier MTBLS7507. It is anticipated that this accession number will be released by 4 June 2023; until that time, the data will be available from the corresponding author upon request.
