# Supplementary material for: Gut Microbiota Taxon-Dependent Transformation of Microglial M1/M2 Phenotypes Underlying Mechanisms of Spatial Learning and Memory Impairment after Chronic Methamphetamine Exposure
Source: Microbiol Spectr. 2023 May 22;11(3):e00302-23. doi: 10.1128/spectrum.00302-23 (PMC10269813; doi:10.1128/spectrum.00302-23)
Supplement: Supplemental file 1 — Fig. S1. Download spectrum.00302-23-s0001.pdf, PDF file, 0.6 MB [file spectrum.00302-23-s0001.pdf]

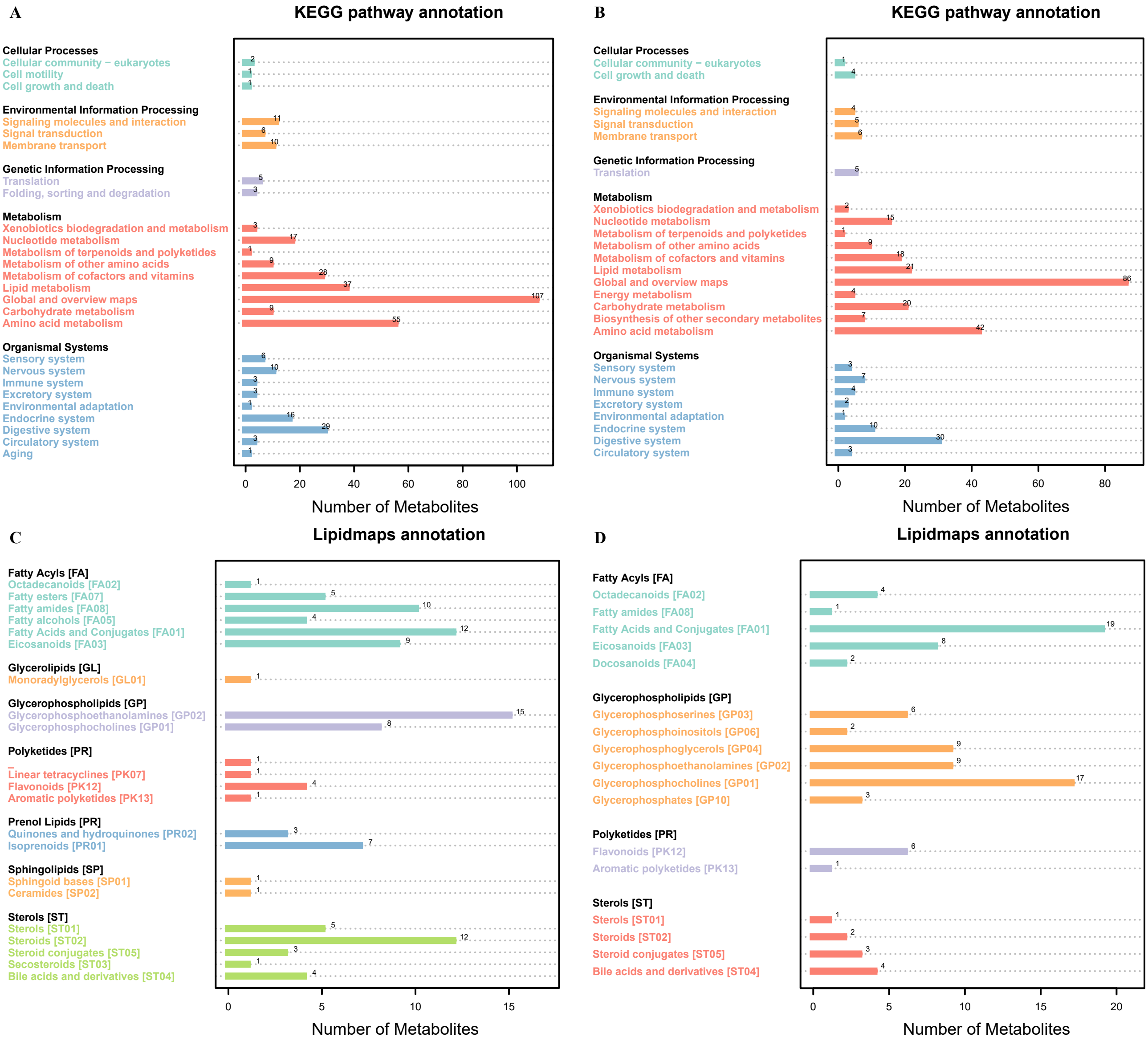
A
KEGG pathway annotation
Cellular Processes
Cellular community − eukaryotes 2
Cell motility 1
Cell growth and death 1
Environmental Information Processing
Signaling molecules and interaction 11
Signal transduction 6
Membrane transport 10
Genetic Information Processing
Translation 5
Folding, sorting and degradation 3
Metabolism
Xenobiotics biodegradation and metabolism 3
Nucleotide metabolism 17
Metabolism of terpenoids and polyketides 1
Metabolism of other amino acids 9
Metabolism of cofactors and vitamins 28
Lipid metabolism 37
Global and overview maps 107
Carbohydrate metabolism 9
Amino acid metabolism 55
Organismal Systems
Sensory system 6
Nervous system 10
Immune system 3
Excretory system 3
Environmental adaptation 1
Endocrine system 16
Digestive system 29
Circulatory system 3
Aging 1
0 20 40 60 80 100
Number of Metabolites
B
KEGG pathway annotation
Cellular Processes
Cellular community − eukaryotes 1
Cell growth and death 4
Environmental Information Processing
Signaling molecules and interaction 4
Signal transduction 5
Membrane transport 6
Genetic Information Processing
Translation 5
Metabolism
Xenobiotics biodegradation and metabolism 2
Nucleotide metabolism 15
Metabolism of terpenoids and polyketides 1
Metabolism of other amino acids 9
Metabolism of cofactors and vitamins 18
Lipid metabolism 21
Global and overview maps 86
Energy metabolism 4
Carbohydrate metabolism 20
Biosynthesis of other secondary metabolites 7
Amino acid metabolism 42
Organismal Systems
Sensory system 3
Nervous system 7
Immune system 4
Excretory system 2
Environmental adaptation 1
Endocrine system 10
Digestive system 30
Circulatory system 3
0 20 40 60 80
Number of Metabolites
C
Lipidmaps annotation
Fatty Acyls [FA]
Octadecanoids [FA02] 1
Fatty esters [FA07] 5
Fatty amides [FA08] 10
Fatty alcohols [FA05] 4
Fatty Acids and Conjugates [FA01] 12
Eicosanoids [FA03] 9
Glycerolipids [GL]
Monoradylglycerols [GL01] 1
Glycerophospholipids [GP]
Glycerophosphoethanolamines [GP02] 15
Glycerophosphocholines [GP01] 8
Polyketides [PR]
Linear tetracyclines [PK07] 1
Flavonoids [PK12] 4
Aromatic polyketides [PK13] 1
Prenol Lipids [PR]
Quinones and hydroquinones [PR02] 3
Isoprenoids [PR01] 7
Sphingolipids [SP]
Sphingoid bases [SP01] 1
Ceramides [SP02] 1
Sterols [ST]
Sterols [ST01] 5
Steroids [ST02] 12
Steroid conjugates [ST05] 3
Secosteroids [ST03] 1
Bile acids and derivatives [ST04] 4
0 5 10 15
Number of Metabolites
D
Lipidmaps annotation
Fatty Acyls [FA]
Octadecanoids [FA02] 4
Fatty amides [FA08] 1
Fatty Acids and Conjugates [FA01] 19
Eicosanoids [FA03] 8
Docosanoids [FA04] 2
Glycerophospholipids [GP]
Glycerophosphoserines [GP03] 6
Glycerophosphoinositols [GP06] 2
Glycerophosphoglycerols [GP04] 9
Glycerophosphoethanolamines [GP02] 9
Glycerophosphocholines [GP01] 17
Glycerophosphates [GP10] 3
Polyketides [PR]
Flavonoids [PK12] 6
Aromatic polyketides [PK13] 1
Sterols [ST]
Sterols [ST01] 1
Steroids [ST02] 2
Steroid conjugates [ST05] 3
Bile acids and derivatives [ST04] 4
0 5 10 15 20
Number of Metabolites

Fig S1 Functional enrichment analysis of differentially expressed metabolites. (A) KEGG pathway enrichment analysis of differential metabolites in ES+. (B) KEGG pathway enrichment analysis of differential metabolites in ES-. (C) Lipidmaps annotation of differential metabolites in ES+. (D) Lipidmaps annotation of differential metabolites in ES-. n = 6 for each group.
