# Supplementary material for: Gut Microbiota Taxon-Dependent Transformation of Microglial M1/M2 Phenotypes Underlying Mechanisms of Spatial Learning and Memory Impairment after Chronic Methamphetamine Exposure
Source: Microbiol Spectr. 2023 May 22;11(3):e00302-23. doi: 10.1128/spectrum.00302-23 (PMC10269813; doi:10.1128/spectrum.00302-23)
Supplement: Supplemental file 2 — Tables S1-S4. Download spectrum.00302-23-s0002.pdf, PDF file, 0.3 MB [file spectrum.00302-23-s0002.pdf]

Supplemental Tables

Table S1. Mediation analysis of microglial M1/M2 phenotype, pro-inflammatory/anti-inflammatory cytokines and the proteins of the BDNF signaling system on the association of Clostridia with spatial learning and memory ability

Mediator	Outcome	Total effect	ACME	Mediation proportion
**M1 microglia and pro-inflammatory cytokines**				
M1	EL	91.2% (52.6%, 122.0%)	47.5% (-4.1%, 117.0%)	47.2%
IL-1 β	EL	86.8% (50.2%, 117.0%)	88.1% (-8.1%, 156.0%)	112.4%
IL-6	EL	86.7% (47.0%, 123.0%)	43.9% (9.4%, 106.0%)	47.9%
M1	NPC	-87.2% (-118.8%, -61.0%)	-23.3% (-73.8%, 33.0%)	22.6%
IL-1 β	NPC	-94.2% (-132.7%, -63.0%)	-52.4% (-132.2%,19.0%)	51.8%
IL-6	NPC	-88.9% (-127.6%, -46.0%)	-13.5% (-62.5%, 21.0%)	12.8%
M1	TSTQ	-90.9% (-133.2%, -59.0%)	-45.6% (-101.7%, 1.0%)	48.5%
IL-1 β	TSTQ	-89.8% (-121.9%, -63.0%)	-79.1% (-140.6%, -9.0%)	85.9%
IL-6	TSTQ	-89.4% (-125.9%, -55.0%)	-24.7% (-64.9%, 16.0%)	30.2%
**M2 microglia, anti-inflammatory cytokines, and the proteins of the BDNF signaling system**				
M2	EL	86.8% (49.2%, 121.0%)	42.9% (-0.4%, 86.0%)	49.2%
IL-4	EL	91.0% (44.3%, 119.0%)	12.5% (-25.0%, 69.0%)	11.0%
IL-10	EL	87.9% (49.7%, 116.0%)	-11.6% (-68.3%, 38.0%)	-6.8%
TGF- β	EL	88.8% (60.3%, 124.0%)	38.2% (-2.1%, 83.0%)	44.6%
mBDNF	EL	87.0% (57.8%, 118.0%)	16.4% (-43.1%, 66.0%)	19.8%
TrkB	EL	91.0% (59.7%, 135.0%)	37.0% (3.3%, 90.0%)	41.7%
proBDNF	EL	88.1% (57.2%, 117.0%)	45.6% (17.0%, 86.0%)	54.7%
$p75^{NTR}$	EL	90.2% (55.3%, 121.0%)	17.6% (-27.7%, 74.0%)	15.9%
t-PA	EL	86.4% (57.0%, 114.0%)	60.2% (13.1%, 127.0%)	70.3%
M2	NPC	-84.2% (-119.3%, -60.0%)	-42.3% (-75.2%, -9.0%)	47.3%
IL-4	NPC	-91.3% (-121.2%, -55.0%)	-3.6% (-38.5%, 34.0%)	1.9%
IL-10	NPC	-88.8% (-125.0%, -62.0%)	15.7% (-23.3%, 56.0%)	-20.6%

TGF- β	NPC	-88.0% (-114.8%, -57.0%)	-42.5% (-108.8%, 1.0%)	45.2%
mBDNF	NPC	-87.4% (-110.8%, -53.0%)	-8.2% (-69.6%, 42.0%)	12.8%
TrkB	NPC	-89.8% (-114.5%, -60.0%)	-14.4% (-55.0%, 42.0%)	17.8%
proBDNF	NPC	-87.4% (-136.2%, -50.0%)	-51.1% (-95.5%, -19.0%)	54.9%
$p75^{NTR}$	NPC	-91.2% (-121.8%, -60.0%)	-21.3% (-79.7%, 21.0%)	18.5%
t-PA	NPC	-88.2% (-118.1%, -58.0%)	-12.8% (-87.1%, 60.0%)	20.4%
M2	TSTQ	-94.5% (-125.1%, -60.0%)	-62.1% (-99.9%, -26.0%)	62.7%
IL-4	TSTQ	-88.7% (-119.7%, -52.0%)	-13.8% (-56.8%, 24.0%)	13.4%
IL-10	TSTQ	-92.7% (-132.7%, -62.0%)	1.9% (-45.2%, 68.0%)	1.3%
TGF- β	TSTQ	-95.9% (-133.9%, -65.0%)	-45.0% (-97.4%, 7.0%)	47.9%
mBDNF	TSTQ	-88.2% (-128.1%, -54.0%)	-16.2% (-87.2%, 30.0%)	14.9%
TrkB	TSTQ	-89.5% (-126.0%, -60.0%)	-33.1% (-78.1%, 15.0%)	39.3%
proBDNF	TSTQ	-92.6% (-134.1%, -55.0%)	-47.0% (-101.8%, -13.0%)	50.9%
$p75^{NTR}$	TSTQ	-87.8% (-128.0%, -50.0%)	-16.3% (-67.5%, 29.0%)	15.5%
t-PA	TSTQ	-87.8% (-116.3%, -57.0%)	-38.6% (-98.4%, 43.0%)	50.8%

Note： Data are shown as percent changes and 95% confidence intervals. EL: escape latency; NPC: number of platform crossings; TSTQ: time spent in the target quadrant (% of total time). EL is the indicator of spatial learning ability. NPC and TSTQ are indicators of spatial memory ability.

Table S2. Mediation analysis of microglial M1/M2 phenotype, pro-inflammatory/anti-inflammatory cytokines and the proteins of the BDNF signaling system on the association of *muribaculaceae* with spatial learning and memory ability

Mediator	Outcome	Total effect	ACME	Mediation proportion
**M1 microglia and pro-inflammatory cytokines**				
M1	EL	-78.6% (-123.2%, -32.0%)	-42.6% (-82.1%, -11.0%)	53.4%
IL-1 β	EL	-83.5% (-132.7%, -47.0%)	-57.5% (-109.7%, -22.0%)	69.0%
IL-6	EL	-85.7% (-136.1%, -49.0%)	-59.4% (-117.4%, -21.0%)	69.2%
TNF- α	EL	-79.3% (-136.9%, -41.0%)	-36.5% (-95.3%, -1.0%)	44.7%
M1	NPC	77.5% (20.4%, 126.0%)	42.3% (2.5%, 78.0%)	55.7%
IL-1 β	NPC	73.1% (31.8%, 143.0%)	62.5% (16.4%, 147.0%)	87.3%
IL-6	NPC	76.3% (38.6%, 112.0%)	50.1% (-18.6%, 117.0%)	62.5%
TNF- α	NPC	71.2% (19.9%, 125.0%)	50.0% (10.3%, 129.0%)	69.0%
M1	TSTQ	73.1% (25.1%, 113.0%)	39.9% (5.7%, 92.0%)	54.6%
IL-1 β	TSTQ	71.6% (24.0%, 113.0%)	59.6% (8.8%, 115.0%)	82.7%
IL-6	TSTQ	76.1% (38.9%, 121.0%)	41.9% (0.3%, 100.0%)	60.2%
TNF- α	TSTQ	72.4% (19.0%, 114.0%)	33.4% (-3.2%, 84.0%)	43.2%
**M2 microglia, anti-inflammatory cytokines, and the proteins of the BDNF signaling system**				
M2	EL	-82.6% (-116.6%, -42.0%)	-43.2% (-84.4%, -11.0%)	50.4%
IL-4	EL	-81.5% (-121.0%, -39.0%)	-28.1% (-70.5%, 2.0%)	33.6%
TGF- β	EL	-79.8% (-122.9%, -43.0%)	-48.9% (-99.0%, -13.0%)	61.2%
mBDNF	EL	-81.0% (-115.9%, -33.0%)	-32.1% (-72.1%, 1.0%)	38.4%
TrkB	EL	-83.7% (-130.2%, -42.0%)	-73.9% (-136.9%, -21.0%)	84.4%
proBDNF	EL	-81.2% (-123.5%, -36.0%)	-79.6% (-165.1%, -28.0%)	99.0%
$p75^{NTR}$	EL	-84.8% (-123.7%, -47.0%)	-22.3% (-61.5%, 19.0%)	23.7%
t-PA	EL	-81.5% (-124.7%, -40.0%)	-43.8% (-81.8%, -9.0%)	53.0%
M2	NPC	66.7% (25.8%, 101.0%)	44.9% (13.4%, 96.0%)	62.8%
IL-4	NPC	75.4% (21.5%, 128.0%)	22.1% (-27.7%, 85.0%)	29.5%

TGF- β	NPC	71.4% (30.9%, 114.0%)	52.8% (10.3%, 119.0%)	69.9%
mBDNF	NPC	81.1% (26.6%, 141.0%)	35.7% (0.2%, 91.0%)	46.1%
TrkB	NPC	73.9% (29.0%, 128.0%)	53.7% (-39.9%, 159.0%)	64.3%
proBDNF	NPC	72.1% (21.6%, 125.0%)	101.8% (57.9%, 192.0%)	144.4%
$p75^{NTR}$	NPC	77.0% (26.8%, 134.0%)	23.5% (-25.0%, 86.0%)	32.9%
t-PA	NPC	74.6% (31.5%, 124.0%)	42.9% (4.8%, 97.0%)	51.7%
M2	TSTQ	79.1% (41.3%, 121.0%)	53.4% (12.2%, 99.0%)	62.6%
IL-4	TSTQ	71.4% (14.6%, 125.0%)	26.3% (-15.9%, 76.0%)	40.2%
TGF- β	TSTQ	81.1% (47.5%, 136.0%)	60.4% (16.0%, 116.0%)	75.5%
mBDNF	TSTQ	71.9% (32.8%, 135.0%)	27.9% (2.8%, 77.0%)	38.7%
TrkB	TSTQ	75.3% (36.6%, 117.0%)	66.5% (-8.9%, 150.0%)	97.7%
proBDNF	TSTQ	83.4% (36.1%, 125.0%)	93.5% (32.1%, 152.0%)	107.4%
$p75^{NTR}$	TSTQ	81.6% (32.6%, 122.0%)	26.2% (-11.0%, 60.0%)	32.2%
t-PA	TSTQ	71.9% (24.5%, 112.0%)	37.8% (3.8%, 71.0%)	48.3%

Note：Data are shown as percent changes and 95% confidence intervals. EL: escape latency; NPC: number of platform crossings; TSTQ: time spent in the target quadrant (% of total time). EL is the indicator of spatial learning ability. NPC and TSTQ are indicators of spatial memory ability.

Table S3. Mediation analysis of microglial M1/M2 phenotype, pro-inflammatory/anti-inflammatory cytokines and the proteins of the BDNF signaling system on the association of *bacteroides* with spatial learning and memory ability

**Mediator**	**Outcome**	**Total effect**	**ACME**	**Mediation proportion**
**M1 microglia and pro-inflammatory cytokines**				
M1	EL	93.0% (63.9%, 128.0%)	74.4% (-40.4%, 186.0%)	82.3%
IL-1 β	EL	88.9% (61.5%, 132.0%)	50.8% (19.9%, 103.0%)	54.8%
IL-6	EL	93.8% (56.7%, 127.0%)	49.5% (7.6%, 96.0%)	50.4%
M1	NPC	-86.9% (-133.5%, -49.0%)	-62.4% (-185.5%, 99.0%)	73.4%
IL-1 β	NPC	-89.5% (-126.1%, -62.0%)	-60.8% (-125.5%, -18.0%)	65.8%
IL-6	NPC	-84.8% (-119.6%, -38.0%)	-27.1% (-90.1%, 45.0%)	31.0%
M1	TSTQ	-88.1% (-130.0%, -50.0%)	-55.2% (-172.9%, 50.0%)	80.2%
IL-1 β	TSTQ	-85.8% (-115.4%, -45.0%)	-51.3% (-84.8%, -22.0%)	58.9%
IL-6	TSTQ	-84.3% (-126.8%, -48.0%)	-24.7% (-74.2%, 48.0%)	26.3%
**M2 microglia, anti-inflammatory cytokines, and the proteins of the BDNF signaling system**				
M2	EL	92.0% (66.4%, 127.0%)	46.8% (1.0%, 98.0%)	51.7%
IL-4	EL	87.4% (45.3%, 127.0%)	14.7% (-17.2%, 46.0%)	17.2%
TGF- β	EL	92.0% (52.5%, 120.0%)	41.0% (7.3%, 103.0%)	39.1%
mBDNF	EL	96.2% (53.8%, 131.0%)	10.5% (-53.6%, 71.0%)	16.9%
TrkB	EL	89.3% (60.7%, 128.0%)	34.4% (8.5%, 75.0%)	34.1%
proBDNF	EL	92.7% (58.4%, 120.0%)	46.1% (18.9%, 85.0%)	49.4%
$p75^{NTR}$	EL	88.5% (58.5%, 134.0%)	22.2% (-10.0%, 78.0%)	24.5%
t-PA	EL	90.6% (53.0%, 125.0%)	66.8% (-12.4%, 147.0%)	80.9%
M2	NPC	-87.8% (-124.9%, -53.0%)	-73.4% (-155.8%, -3.0%)	86.8%
IL-4	NPC	-82.0% (-115.6%, -35.0%)	-17.0% (-69.6%, 33.0%)	21.4%
TGF- β	NPC	-83.8% (-116.7%, -42.0%)	-52.2% (-97.5%, -13.0%)	62.7%
mBDNF	NPC	-86.9% (-125.4%, -49.0%)	-17.7% (-107.9%, 71.0%)	17.3%
TrkB	NPC	-82.8% (-122.6%, -50.0%)	-28.1% (-90.0%, 35.0%)	32.0%

proBDNF	NPC	-88.2% (-118.5%, -46.0%)	-50.6% (-85.5%, -18.0%)	58.8%
p75^{NTR}	NPC	-84.9% (-114.1%, -47.0%)	-36.9% (-105.5%, -2.0%)	40.9%
t-PA	NPC	-81.9% (-123.0%, -47.0%)	-32.0% (-148.4%, 71.0%)	26.6%
M2	TSTQ	-90.3% (-144.2%, -58.0%)	-74.9% (-142.0%, -34.0%)	82.1%
IL-4	TSTQ	-90.7% (-122.8%, -56.0%)	-19.6% (-76.5%, 16.0%)	18.4%
TGF- β	TSTQ	-92.7% (-126.1%, -58.0%)	-43.9% (-86.4%, -14.0%)	43.5%
mBDNF	TSTQ	-91.0% (-130.8%, -59.0%)	-9.5% (-66.5%, 59.0%)	9.9%
TrkB	TSTQ	-86.8% (-123.3%, -49.0%)	-35.9% (-82.3%, -6.0%)	43.2%
proBDNF	TSTQ	-86.7% (-125.7%, -47.0%)	-44.2% (-75.5%, -12.0%)	49.9%
p75^{NTR}	TSTQ	-87.2% (-122.2%, -59.0%)	-19.8% (-61.5%, 9.0%)	22.3%
t-PA	TSTQ	-89.4% (-122.5%, -52.0%)	-39.9% (-130.9%, 63.0%)	51.6%

Note： Data are shown as percent changes and 95% confidence intervals. EL: escape latency; NPC: number of platform crossings; TSTQ: time spent in the target quadrant (% of total time). EL is the indicator of spatial learning ability. NPC and TSTQ are indicators of spatial memory ability.

Table S4. Mediation analysis of microglial M1/M2 phenotype, pro-inflammatory/anti-inflammatory cytokines and the proteins of the BDNF signaling system on the association of *lactobacillus* with spatial learning and memory ability

Mediator	Outcome	Total effect	ACME	Mediation proportion
**M1 microglia and pro-inflammatory cytokines**				
M1	EL	88.9% (51.8%, 115.0%)	62.9% (3.4%, 147.0%)	69.2%
IL-1 β	EL	91.5% (66.1%, 125.0%)	67.2% (14.8%, 112.0%)	71.4%
IL-6	EL	90.2% (54.5%, 128.0%)	49.4% (-30.7%, 146.0%)	53.6%
M1	NPC	-79.7% (-126.1%, -34.0%)	-90.2% (-215.5%, 17.0%)	116.7%
IL-1 β	NPC	-80.9% (-122.4%, -45.0%)	-86.8% (-173.7%, -26.0%)	107.6%
IL-6	NPC	-78.3% (-116.8%, -22.0%)	-35.6% (-146.7%, 108.0%)	59.9%
M1	TSTQ	-87.9% (-137.5%, -47.0%)	-77.8% (-160.4%, 11.0%)	90.2%
IL-1 β	TSTQ	-86.2% (-124.8%, -49.0%)	-83.6% (-152.4%, -22.0%)	98.8%
IL-6	TSTQ	-83.0% (-118.9%, -37.0%)	-5.4% (-96.3%, 86.0%)	10.8%
**M2 microglia, anti-inflammatory cytokines, and the proteins of the BDNF signaling system**				
M2	EL	88.6% (53.6%, 120.0%)	43.9% (9.7%, 81.0%)	48.4%
IL-4	EL	92.1% (65.6%, 128.0%)	16.4% (-28.2%, 65.0%)	14.6%
TGF- β	EL	94.1% (58.3%, 134.0%)	44.7% (2.5%, 96.0%)	49.2%
mBDNF	EL	85.4% (58.8%, 107.0%)	23.1% (-20.6%, 78.0%)	29.4%
TrkB	EL	88.8% (51.1%, 129.0%)	36.8% (-9.0%, 98.0%)	38.9%
proBDNF	EL	88.4% (49.0%, 120.0%)	49.9% (20.8%, 87.0%)	55.9%
$p75^{NTR}$	EL	93.5% (60.9%, 126.0%)	25.5% (-31.2%, 73.0%)	28.4%
t-PA	EL	93.1% (57.3%, 129.0%)	60.6% (-5.9%, 144.0%)	66.0%
M2	NPC	-77.9% (-115.3%, -30.0%)	-58.6% (-94.9%, -8.0%)	75.7%
IL-4	NPC	-77.8% (-119.5%, -31.0%)	-25.2% (-88.0%, 16.0%)	26.4%
TGF- β	NPC	-78.8% (-125.3%, -36.0%)	-69.1% (-153.4%, -14.0%)	90.8%
mBDNF	NPC	-75.4% (-126.2%, -30.0%)	-38.3% (-131.0%, 17.0%)	44.6%
TrkB	NPC	-79.9% (-127.4%, -22.0%)	-35.5% (-112.5%, 25.0%)	37.3%

proBDNF	NPC	-75.9% (-118.1%, -34.0%)	-59.6% (-115.0%, -15.0%)	80.2%
$p75^{NTR}$	NPC	-83.4% (-122.2%, -43.0%)	-40.9% (-96.3%, 21.0%)	51.5%
t-PA	NPC	-73.1% (-120.2%, -22.0%)	-49.1% (-150.0%, 27.0%)	54.7%
M2	TSTQ	-89.8% (-131.5%, -39.0%)	-70.2% (-124.5%, -23.0%)	78.9%
IL-4	TSTQ	-87.1% (-130.7%, -42.0%)	-17.4% (-68.7%, 42.0%)	21.1%
TGF- β	TSTQ	-88.8% (-130.5%, -54.0%)	-55.4% (-103.1%, 1.0%)	62.6%
mBDNF	TSTQ	-83.3% (-130.2%, -35.0%)	-27.4% (-101.9%, 36.0%)	27.1%
TrkB	TSTQ	-88.0% (-140.0%, -43.0%)	-48.6% (-111.5%, -3.0%)	58.9%
proBDNF	TSTQ	-82.7% (-121.5%, -46.0%)	-52.4% (-89.8%, -18.0%)	65.5%
$p75^{NTR}$	TSTQ	-81.6% (-126.6%, -43.0%)	-32.0% (-91.5%, 18.0%)	37.7%
t-PA	TSTQ	-88.3% (-134.1%, -52.0%)	-58.4% (-158.9%, 15.0%)	58.7%

Note： Data are shown as percent changes and 95% confidence intervals. EL: escape latency; NPC: number of platform crossings; TSTQ: time spent in the target quadrant (% of total time). EL is the indicator of spatial learning ability. NPC and TSTQ are indicators of spatial memory ability.
